# Proprotein Convertases and the Complement System

**DOI:** 10.3389/fimmu.2022.958121

**Published:** 2022-07-06

**Authors:** József Dobó, Andrea Kocsis, Ráhel Dani, Péter Gál

**Affiliations:** Institute of Enzymology, Research Centre for Natural Sciences, Budapest, Hungary

**Keywords:** complement system, proprotein convertase, lectin pathway, alternative pathway, classical pathway, protein secretion and processing, MASP-3

## Abstract

Proteins destined for secretion - after removal of the signal sequence - often undergo further proteolytic processing by proprotein convertases (PCs). Prohormones are typically processed in the regulated secretory pathway, while most plasma proteins travel though the constitutive pathway. The complement system is a major proteolytic cascade in the blood, serving as a first line of defense against microbes and also contributing to the immune homeostasis. Several complement components, namely C3, C4, C5 and factor I (FI), are multi-chain proteins that are apparently processed by PCs intracellularly. Cleavage occurs at consecutive basic residues and probably also involves the action of carboxypeptidases. The most likely candidate for the intracellular processing of complement proteins is furin, however, because of the overlapping specificities of basic amino acid residue-specific proprotein convertases, other PCs might be involved. To our surprise, we have recently discovered that processing of another complement protein, mannan-binding lectin-associated serine protease-3 (MASP-3) occurs in the blood by PCSK6 (PACE4). A similar mechanism had been described for the membrane protease corin, which is also activated extracellularly by PCSK6. In this review we intend to point out that the proper functioning of the complement system intimately depends on the action of proprotein convertases. In addition to the non-enzymatic components (C3, C4, C5), two constitutively active complement proteases are directly activated by PCs either intracellularly (FI), or extracellularly (MASP-3), moreover indirectly, through the constitutive activation of pro-factor D by MASP-3, the activity of the alternative pathway also depends on a PC present in the blood.

## Synopsis of The Complement System

**Figure 1:**
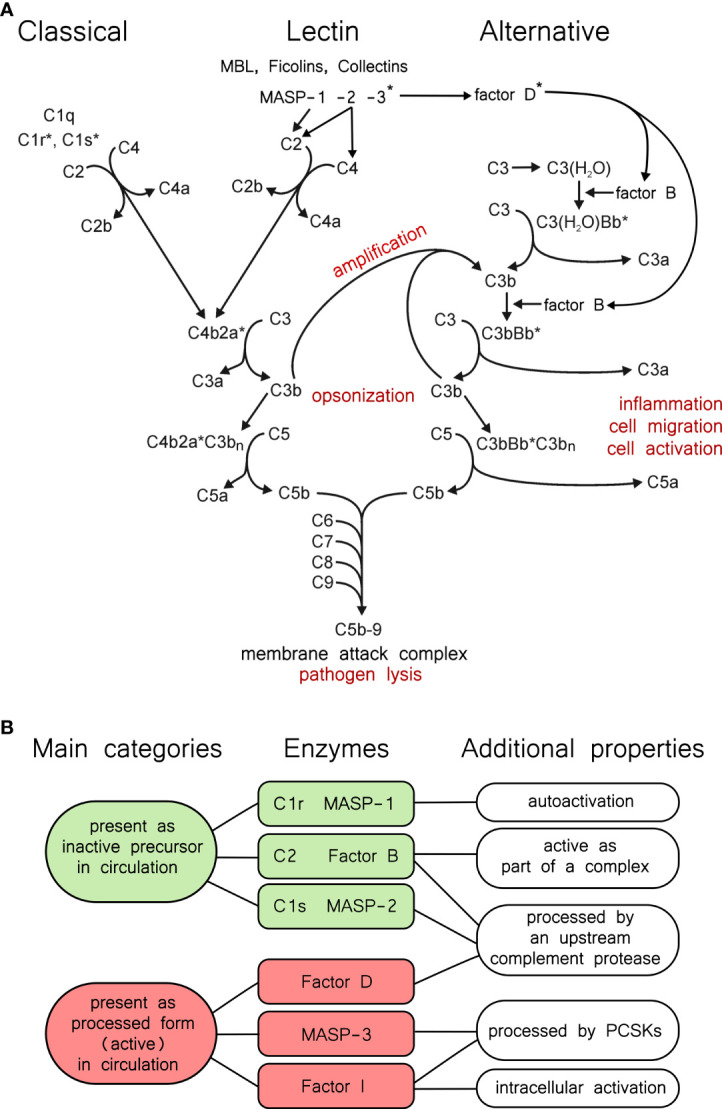
The complement system and properties of its proteases. **(A)** Overview of the complement system. The three activation routes, the amplification loop, and the terminal pathway are described in the text, and this panel is intended to aid the reader as a guide. Asterisks indicate active proteases. **(B)** Categories of complement proteases by activation status. Green and red shading indicates the two main categories. Typical serine proteases are synthesized in the proenzyme form, and remain proenzymic until a they are cleaved into the active form following a trigger. In the complement system C1r, C1s, MASP-1, and MASP-2 fall into this category. Among these, C1r and MASP-1 can efficiently autoactivate. MASP-3, FD, and FI, are present predominantly (or completely) in the active form in the blood, however they have very narrow substrate specificities. C2 and FB are special cases, because they gain a transient activity following cleavage as parts of the C4bC2a, or the C3bBb complex.

The complement system (**Figure 1A**) is an integral part of the immune system. It is the most powerful molecular effector arm of the innate immune system, but it is also connected to the adaptive immunity in many ways [for reviews see (1–4)]. The most important components of the complement system are serine proteases (**Figure 1B**), which form a proteolytic cascade system (5). Other components include pattern recognition molecules (C1q, MBL, ficolins), the thioester-containing molecules (C3, C4), the components of the membrane attack complex (C5, C6, C7, C8, C9), membrane-bound receptors and regulators, and fluid-phase regulators. These proteins act in synergy to monitor surfaces, and to label and eliminate invading pathogen microbes or damaged or altered self-cells. Because of the cascade-like manner of complement activation the initial activation signal is amplified tremendously and results in a highly efficient cytotoxic effect and in releasing potent inflammation-promoting proteolytic fragments (e.g. anaphylatoxins). The activity of the complement system is regulated in many ways to avoid self-tissue damage and to focus the complement-mediated damage to the pathogens. Uncontrolled, excessive activation of the complement system could result in or contribute to the development of many serious diseases [for reviews see (6–8)].

Depending on the activation-surface the complement cascade can be activated through three different pathways: the classical, the lectin, and the alternative pathways (**Figure 1A**). Immune complexes are the most powerful activators of the classical pathway. C1q binds to the IgG or IgM components of the immune complexes which results in the activation of the C1r and C1s serine proteases (9, 10). One C1q molecule with two C1r and two C1s molecules form the C1 complex (11). C1s is the executive protease of the C1 complex. It cleaves the subsequent components of the classical pathway: C4 and C2. The bigger proteolytic fragment of C4, C4b binds to the surface through an ester or amid bound and captures C2, the serine protease component of the classical pathway C3-convertase enzyme complex. C4b-bound C2 is cleaved and activated by C1s, yielding the C4bC2a complex.

The activation of the lectin pathway generates the same C3 convertase complex as the classical pathway. In the lectin pathway there are various pattern recognition molecules (MBL, ficolins, collectins) that are associated with the serine protease components MASPs (12). Upon the pattern recognition molecules bind to the carbohydrate pattern on the surface of the bacteria, MASP-1 autoactivates and activates MASP-2 (13). MASP-2 cleaves C4, while C2 is cleaved by both MASP-1 and MASP-2 generating the C4b2a convertase complex. Regardless of the activation route, C3 is cleaved by the C3 convertase complexes into two pieces. The bigger fragment, C3b binds to the activation surface like C4b, while the smaller fragment, the anaphylatoxin C3a is released to the circulation.

The alternative pathway initiates when C3b appears on the activation surface (14). C3b captures FB and the C3b-bound FB is cleaved by FD. FD is continuously cleaved by MASP-3 in the blood, even before the advent of any activation signal. C3bBb is the alternative pathway C3 convertase, which cleaves more C3b resulting in generation of more C3 convertase complexes. This positive feed-back mechanism ensures the amplification of the complement activation initiated by either the classical or the alternative pathway (15). The alternative pathway can also be initiated on its own through the so-called “tick-over” mechanism (16). C3 slowly hydrolyses in the circulation and the resulting C3(H_2_O) resembles to C3b. C3(H_2_O) binds FB, which is cleaved by FD. C3(H_2_O)Bb is a fluid phase C3 convertase which can generate C3b near any surface. The surfaces of the self-cells are protected by different complement inhibitors against complement-mediated damage. On the unprotected surfaces (e.g. bacterial surface), however, the deposited C3b initiates the amplification loop of the alternative pathway leading to full-scale complement activation.

When the density of the deposited C3b reaches a certain point the specificity of the C3 convertase complexes switches to C5 (17, 18), which are traditionally regarded as the C5 convertases (C4b2aC3b_n_, C3bBbC3b_n_). From this point the three activation pathways continue in a common terminal pathway. The smaller proteolytic fragment of C5, C5a is an extremely potent anaphylatoxin. The larger proteolytic fragment, C5b, binds C6 and C7. The C5b-7 complex associates with the membrane of the invaded cells and captures C8 and multiple C9 molecules (19). After conformational changes the C5b-9 complex forms a pore on the cell membrane resulting in the lysis and destruction of the target cell.

Since the complement system is potentially dangerous to the host, there are various mechanisms to control complement activity. The activity of the serine proteases of the complement system is regulated by two ways. The first way is zymogen activation. Most of the serine proteases of the complement system are synthetized and secreted as inactive zymogens (5). Activation during the cascade reaction occurs through limited proteolysis. The one-chain zymogen molecule is cleaved at the activation peptide, and the resulting two-chain molecule has the full proteolytic activity. The logic behind the amplification power of the proteolytic cascade systems lies in that each active serine protease cleaves and activates multiple zymogen molecules downstream the cascade. In this way an exponential amplification scheme ensures the efficient answer against the initial stimulus. The other proteolytic cascade systems in the blood (e.g. blood coagulation, fibrinolysis) work on the same principle (20). These cascade systems are evolutionary and functionally closely related and there are many cross-talks between them. Probably the most interesting step in the cascade reaction is the first proteolytic step, which is an autoactivation in the case of the classical and the lectin pathway. In this case the “inactive” zymogen molecule has some low proteolytic activity, that is enough to activate another zymogen molecule. The resulting active protease then activates more zymogen molecules with high efficiency. C1r in the classical pathway and MASP-1 in the lectin pathway have pronounced autoactivation capacity (**Figure 1B**). MASP-2 also can autoactivate, however, it takes place only in high concentration and after prolonged incubation (21). This is not the physiological case; in order to get a quick and efficient lectin pathway activation MASP-1 autoactivates and cleaves zymogen MASP-2 (13). Interestingly, active MASP-2 cleaves zymogen MASP-1 more efficiently, than zymogen MASP-2 (22). Zymogen C1s has no autoactivation ability, it is cleaved exclusively by C1r. The serine proteases of the C3/C5 convertase complexes, C2 and FB, also cannot autoactivate. C2 is activated by C1s, MASP-1, or MASP-2, while FB is activated by FD. Surprisingly, there are three serine proteases of the complement system which are present in cleaved, processed form in the circulation: MASP-3, FI and FD (**Figure 1B**). As we will see in the following sections these proteases are processed quite differently. MASP-3 and FD are present predominantly in cleaved form in the blood, however zymogen molecules were also detected in low concentrations (23, 24). FI is fully converted. In the case of these proteases proteolytic processing alone does not ensure proteolytic activity. In the case of the FD and FI the cleaved molecules do not have the conformation necessary for proteolytic activity (25, 26). They are present in a distorted conformation, and the fully active conformation is induced by the substrate. These proteases have very narrow substrate specificity. FD cleaves only C3b-bound FB. The proconvertase complex, C3bB, binds FD and induces the conformational change in the molecule to get full proteolytic activity (27). FI can cleave C3b and C4b but only in the presence of cofactors (FH for C3b, C4BP for C4b, MCP or CR1 for both). FI gains the proteolytic conformation in the C3b/C4b-cofactor-FI ternary complex (28). There is no experimental 3D structure of empty activated MASP-3 protease, but we cannot rule out that its structure is distorted, like that of FD and FI. The only proven physiological substrate of MASP-3 is pro-FD. Another substrate might exist, since MASP-3 has a role in the development. Lack of MASP-3 activity results in the development of 3MC (Malpuech-Michels-Mingarelli-Carnevale) syndrome (29, 30).

Another way of regulation is mediated by inhibitors. The most important fluid-phase inhibitors of the serine proteases of the serum cascade systems are the serpins (31, 32). C1-inhibitor inhibits C1r, C1s, MASP-1 and MASP-2 (33). The serpins are “suicide-inhibitors”; they make stable covalent complex with the active serine proteases and prevents them to cleave more substrate. The serpin-serine protease complex is then removed from the circulation. Antithrombin is a major inhibitor of thrombin, but it is an efficient inhibitor of MASP-1, as well, especially in the presence of heparin (34). Intriguingly, the three “pre-cleaved” proteases, MASP-3, FD and FI, have no known physiological inhibitor. These proteases are regulated through their narrow substrate specificity and through the substrate induction. Active C2 (C2a) and FB (Bb) also lack fluid phase inhibitors. The convertases are labile complexes; C2a and Bb can easily dissociate from them. Once dissociated, C2a and Bb cannot re-associate with C4b and C3b, respectively. The dissociation is facilitated by different factors on the cell membrane (35). Interestingly there is a positive regulator of the complement activation, properdin, which stabilizes the alternative pathway C3 convertase (C3bBb), but not the classical/lectin pathway C3 convertase (C4b2a) (36, 37).

It is also important from a regulatory point of view that the activated complement proteins react very quickly before inactivation occurs. This mechanism ensures, that the complement activation takes place locally, preferably on the surface of the pathogens, and not systemically. For example, C3 and C4 contain a reactive thioester bond. After convertase-mediated cleavage, the thioester bond becomes exposed and makes an ester or amid bond on the surface of the target cell (38). If the C3b or C4b cannot reach the cell surface in time, it will react with water (hydrolysis) and cannot attach to surface any more. In this way only the target cell will be destroyed by the complement-mediated attack and the probability of the bystander lysis will be very low. The formation of the membrane attack complex is also regulated. Vitronectin is competing with the membrane binding of C5b-7 complex and prevents its insertion into the membrane (39). Clusterin is also a regulator of membrane attack complex formation (40).

Most of the complement components are synthetized in the liver. There are several exceptions: C1q and properdin are synthesized mainly by leucocytes, FD is expressed almost exclusively by adipose tissue (41). It is interesting that MASP-1 and MASP-3 are the alternative splice products of the *MASP1* gene, but their expression pattern is different. MASP-1 is produced in the liver, while MASP-3 expression was also detected in several extra-hepatic tissues, such as colon, heart and skeletal muscle (42). Local production of complement proteins can also occur in various other tissues including the eye and the kidney, which may contribute to complement-mediated diseases affecting these organs (43, 44).

## Brief Introduction To Mammalian Proprotein Convertases

The story of proprotein convertases (PCs) goes back to the proteolytic processing of pro-insulin, and the discovery of the homology between the yeast protease kexin (KEX2) and its mammalian counterpart furin (45, 46). In this paper we do not wish to review proprotein convertases comprehensively, because there are excellent reviews published in this subject e.g (47–50).. On the other hand, we intend to provide a brief list of the human (mammalian) enzymes (**Table 1**), their function, tissue distribution, and substrate specificity, because it will aid us to categorize PCs that may be required for the processing of certain complement proteins.

**Table 1:** Human (mammalian) proprotein convertase genes and major protein variants.

gene	protein name PCSK nomenclature	other common protein name(s)	intracellular lumenal (L), membrane-bound (M), or secreted (S)	cleaves after basic (B), or non-basic (N) residues	notes
*PCSK1/NEC1*	PCSK1	PC1, PC3, PC1/3, NEC1	L	B	confined to the neuroendocrine system, involved in the processing of proinsulin and other hormones
*PCSK2/NEC2*	PCSK2	PC2, NEC2	L	B	
*PCSK3/FURIN*	PCSK3	furin, PACE	M	B	ubiquitous, highly expressed in the liver
*PCSK4*	PCSK4	PC4	L	B	restricted to testicular and ovarian germ cells
*PCSK5*	PCSK5 short	PC5A, PC6A	S	B	ubiquitous
	PCSK5 long	PC5B, PC6B	M	B	ubiquitous, may have a redundant role with furin
*PCSK6*	PCSK6	PACE4, SPC4	S	B	ubiquitous
*PCSK7*	PCSK7	PC7, PC8	M	B	ubiquitous, may have a redundant role with other PCs
*PCSK8/MBTPS1/SKI1*	PCSK8 (rarely used)	MBTPS1, SKI-1	M	N	ubiquitous, involved in the processing of transcription factors
*PCSK9*	PCSK9	PC9, NARC-1	S	N	cleaves only itself, regulates cholesterol level

It had been known that the two-chain mature insulin, held together by disulfide bridges, is synthetized as a single chain polypeptide pre-pro-insulin (45, 47, 51). After removal of the signal peptide, the first cleavage is performed by PC1 (aka PC3) and the second by PC2 in the secretory granules. The remaining polybasic residues are removed by carboxypeptidases. PC1 and PC2 are confined to neuroendocrine and endocrine cells, where they act as the major processing enzymes in the regulated secretory pathway of peptide hormones. Because of their roles in pro-hormone processing PCs were initially called prohormone convertases. On the other hand, because of their location PC1 and PC2 are unlikely to act on complement proteins *in vivo*, and this is also true for PC4 (PCSK4).

The first mammalian PC to be identified was furin, when it became evident that it is homologous to the yeast enzyme kexin. The serine protease domain of furin and kexin belongs to the subtilisin fold, giving rise to the name: proprotein convertase subtilisin/kexin (PCSK). Furin is probably the best characterized of all PCs. It preferentially cleaves after R-X-K/R-R↓ sequence (where X represents any amino acid), but often it cleaves after the less optimal R-X-X-R↓, or K/R-X-X-X-K/R-R↓ sequences (48). Trafficking of furin is also well characterized, and serves as an example for other PCs. After translocation to the endoplasmic reticulum (ER) and removal of the signal peptide it is anchored to the cell membrane through its C-terminal transmembrane segment. The first autocatalytic cleavage occurs in the ER at neutral pH between the pro-domain and the serine protease domain. The prodomain still associates with the serine protease domain inhibiting furin. The second, slower cleavage occurs within the prodomain at moderately acidic pH in the trans-Golgi network (TGN), secretory vesicles and endosomes, resulting in a fully active enzyme (48, 52). Most furin substrates are processed in these compartments during the constitutive secretory pathway. Membrane bound furin also appears on the cell surface, where it can process several substrates, e.g. viral proteins (50). Furin recycles between the cell surface and the TGN *via* early endosomes. Soluble extracellular furin has been described *via* shedding, and furin can also act in the secretory granules of the regulated secretory pathway. To sum up, furin is active in many compartments, therefore it is generally considered as the major proprotein processing enzyme.

In this review we will use the abbreviation of BAR-PC (basic amino acid residue-specific proprotein convertase), having the general recognition sequence of K/R-X_n_-K/R↓ (where n=0,2,4,6), which typically, but not always contains consecutive (paired) basic amino acid residues (53). Seven genes encode for BAR-PCs and two for PCs that cleave after non-basic residues (**Table 1**). BAR-PCs recognize quite similar sequences therefore some degree of redundancy is possible between the members. In addition to the slightly different preferred sequences, their intracellular trafficking, differential expression in tissues, and the site of their activation can be the determining factors for the substrate specificity of a certain BAR-PC *in vivo*. In general, spatial segregation largely determines the unique features of PCs (54). It is important to note that PCs, like other enzymes of the subtilisin fold are strictly Ca^2+^-dependent enzymes. This property can help differentiate their action from serine proteases of the chymotrypsin fold.

We have started work on PCs because we realized that activation of certain enzymes by PCs can occur in the blood (see later). In the next section we introduce the three known secreted PCs that are present in extracellular fluids. PCSK9 is also included, as a well-known example of a PC present at high levels in the blood, despite being in a proteolytically inactive (inhibited) state. PACE4 has been also detected in the blood (55, 56), while so far no one reported the presence of PC5A in the blood, however it could be present in other extracellular fluids.

## Secreted Proprotein Convertases

### PCSK6 (PACE4)

**Figure 2:**
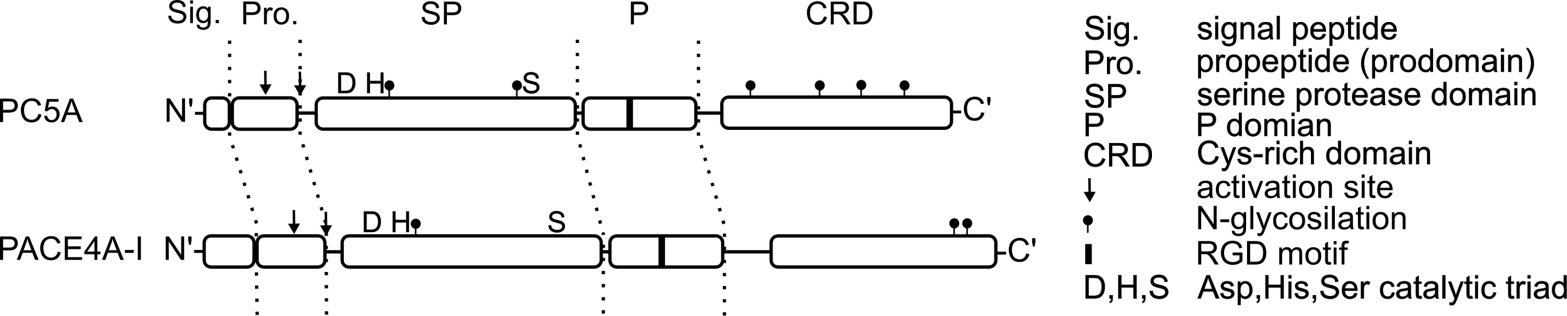
Domain structures of PCSK5 (PC5A isoform), and PCSK6 (PACE4A-I isoform). PC5A is the shorter, secreted isoform of the two main isoforms of PCSK5. PCSK6 (PACE4) has several secreted isoforms. PACE4A-I is considered as the main (canonical) isoform. Two autocatalytic cleavages (↓) after and within the prodomain are required for activation. The second cleavage is thought to occur extracellularly.

*PCSK6* (proprotein convertase subtilisin/kexin type 6) gene is located close to the *FURIN* (*PCSK3*) gene on human chromosome 15, referring to the common origin of these enzymes (57). Eight different splice variants of the *PCSK6* gene have been described, although most of them were found only at mRNA level. The PACE4 (paired basic amino acid cleaving enzyme 4), SPC4 (subtilisin-like proprotein convertase 4), and the more recent PCSK6 names are used simultaneously for the protein products. PACE4 is widely expressed in human tissues. It is mainly expressed in liver, but also in the lung, gut, spleen, brain, placenta and neuroendocrine tissues (58). Three isoforms, PACE4A-I, PACE4A-II, PACE4B are probably secreted *via* the constitutive pathway (59). PACE4 efficiently cleaves substrates at R-X-K/R-R↓, R-X-X-R↓ sequences, and also recognizes short R-R↓ and K-R↓ motifs (60). PACE4A-I is the full-length enzyme (**Figure 2**), and like all PCs requires Ca^2+^ for the catalysis, and also shows marked temperature sensitivity (61). PACE4 has a multidomain structure, before the structural domains, it starts with an extremely long signal sequence (63 amino acids), which is followed by a propeptide (or prodomain), an S8-type serine protease domain, a P (or Homo B) domain, and finally a Cys-rich domain (CRD) composed of tandem furin-like repeats at the C-terminus. PACE4 is synthesized as a zymogen, and its autoactivation requires two proteolytic events after and within the prodomain. The first cleavage takes place in the endoplasmic reticulum (ER) (62), and the second occurs presumably at the cell surface after the secretion (63). In subtilisin-like enzymes, the prodomain acts as an intramolecular chaperone (64), promoting the formation of the correct fold, while its pH-dependent binding to the catalytic domain may inhibit the activity during secretion, as it has been shown for furin (65). The P domain is also essential for appropriate folding, it stabilizes the protease domain through hydrophobic interactions (66) and it carries an RGD tripeptide motif that controls the protein trafficking through the ER (67), and it can also play a role in the binding of integrins on the cell surface. Unlike many other proprotein convertases (PCs) PACE4 lacks the transmembrane region, therefore it is released into the extracellular matrix. A cationic region of the CRD can tether PACE4 to the extracellular matrix on the cell surface *via* heparan sulfate proteoglycans (HSPGs) (68), and there can inactivate the HSPG-bound endothelial lipase and lipoprotein lipase (69) modifying the high-density lipoprotein (HDL) metabolism. CRD also interacts with tissue inhibitors of metalloproteases (TIMPs) (70), and through them PACE4A-I could regulate extracellular matrix remodeling. A quarter of the *PCSK6* knock out mice embryos die prenatally showing sever cardiovascular and craniofacial malformations. Half of the embryos showed left pulmonary isomerism and abnormal, right-sided stomach, pancreas and spleen. Dysfunction of PACE4A-I results an altered left-right patterning during the embryogenesis. PACE4 is responsible for the processing of Nodal precursor protein that activates the transforming growth factor-β (TGF-β) signaling pathway (58, 71). PACE4A-I selectively cleaves the B isoform of insulin receptor at insulin-target tissues that increases the production of glucokinase and affects glucose metabolism (72). PACE4A-I also processes the insulin-like grow factor-II (IGF-II) (73). RNA silencing of PACE4 suppresses the myosin light chain expression in skeletal muscle cells. It could be restored by addition of processed IGF-II suggesting that PACE4A-I accelerates the myogenic differentiation (74). PACE4A-I seems to enhance proliferation of prostate cancer cells *in vitro* and the growth of tumor xenografts in mice (75). In the case of mice keratinocyte cultures PACE4A-I overexpression enhances the invasiveness of malignant cells and it also triggers the conversion of non-invasive cell types to malignant *via* activating matrix metalloproteases (76). In contrast to normal thyroid parenchyma, malignant thyroid nodules expresses PACE4A-I (77). These results suggest that PACE4 could be a promising diagnostic tool and therapeutic target of some cancer types. PACE4A-II isoform has a short deletion in a disordered region after the P domain. It was demonstrated *in vitro* that PACE4A-II processes pro-von Willebrand factor as well as PACE4A-I, and it activates rat complement pro-C3 more efficiently than the full-length form (78).

### PCSK5 (PC5A Isoform)

A PC encoded by the *PCSK5* (proprotein convertase subtilisin/kexin type 5) gene was discovered in two laboratories at the same time (79, 80). It explains why it has two parallel names in the literature, proprotein convertase 5 and 6 (PC5, PC6), but often referred to as PC5/6 or PCSK5. The *PCSK5* gene encodes two protein isoforms PC5A and B having different tissue distribution and structural differences at the C-terminus. Both isoforms are expressed equally in the liver; PC5A is the dominant isoform in heart, lung, ovary and in neuronal tissues, whereas PC5B is the major form in kidney and in the intestinal tract (81). PC5A isoform (**Figure 2**) has a shorter CRD than that of B isoform and it lacks the transmembrane domain, therefore PC5A is soluble and it is secreted on the regulated secretory pathway (82), while PC5B localized only in the trans-Golgi vesicles (83). PC5A shares many structural and *in vitro* functional similarities with PACE4. Like PACE4, it also takes part in regulation of HDL level through inactivation of endothelial lipase and lipoprotein lipase (69). In neural tissues PC5A expression overlaps with the expression of neural adhesion molecule L1. In the hippocampus PC5A performs the first proteolytic cleavage of L1 on the neuron surface which is required for release, dimerization and activation of L1 (84). Through L1 processing PC5A plays a role in neuronal migration, growth and regeneration. Normal *PCSK5* gene function seems to be essential in embryonic development as demonstrated by knock-out studies. Lack of functional *PCSK5* leads to early embryonic death in mice model (81). KO embryos exhibited homeotic malformations, absence of tail and kidneys, development of additional ribs, and collapsed lung. Very similar phenotypes were observed in growth/differentiation factor 11 (Gdf11) knock out mice. PC5 and Gdf11 mRNA showed an overlapped appearance in PCSK KO embryos, suggesting that Gdf11 is a potential substrate of PC5 (85). In human umbilical vein endothelial cells (HUVECs) PC5 is the major activator of receptor protein tyrosine phosphatase μ (RPTPμ) that promotes the monolayer formation of the endothelium based on inhibition of cell growth (86). Separate overexpression of different PCs in colorectal cancer cell line revealed that PC5A is the most potent proteolytic activator of αv-integrin (87). In vascular smooth muscle cells (VSMCs) PC5 activated αv-integrin promotes the cell migration and adhesion to vitronectin (88). A potent vessel remodeling factor, the platelet-derived growth factor (PDGF) upregulates *PCSK5* gene expression in VSMC culture and increases the PC5 protein level, as it was proven *ex vivo* in an atherosclerotic aorta model (89). Co-localization of vitronectin, αv-integrin and PC5 on VSMC in atherosclerotic lesions suggests that PC5 has an important role is the development of atherosclerosis. *In vitro* analyses of PC5 mutants having altered post translational modification (90) and studies of liver specific PC5 knock out mice (91) revealed that PC5A and furin can cleave PCSK9 on hepatocyte surface to modify its fold and activity.

### PCSK9

Based on the observation that subtilisin/kexin isozyme 1 (SKI-1) encoded by the *PCSK8* gene cleaves after non-basic residues, gene databases were screened to identify ortholog enzymes. This *in silico* method led to the discovery of proprotein convertase subtilisin/kexin type 9 (PCSK9), which was first described as neural apoptosis-regulated convertase (NARC-1). PCSK9 mRNA was detected in the liver, kidney, cerebellum, and small intestine. PCSK9 is a member of the proteinase K subfamily. Like other PCs, PCSK9 is synthesized as a zymogen, and the autocatalytic cleavage takes place in the ER. PCSK9 prefers hydrophobic residues at the P1, P3, P5 positions in the recognition motif (92). Compared to the above mentioned PCs, PCSK9 shows structural differences; it lacks the conserved P domain and carries a Cys- and His-rich domain (CHRD) at the C-terminus. In the absence of the P domain, the CHRD domain could stabilize folding (93). After autoactivation, the prodomain remains bound to the catalytic domain locking the substrate-binding groove, thus PCSK9 does not exhibit enzyme activity against any other substrate (94). *PCSK9* gene point mutations are found to be associated with autosomal dominant hypercholesterolemia (95) predicting a role in lipid metabolism. CRHD binds the low-density lipoprotein receptor (LDLR) on hepatocyte surface, enhances its uptake in clathrin coated vesicles and degradation in lysosomes, therefore high PCSK9 level results in increased LDL level in the circulation (96, 97). PCSK9 repression seems to be a promising therapeutic strategy in hypercholesterolemia and in associated cardiovascular diseases.

## Complement Proteins Processes By Proprotein Convertases and The Story of Corin

### C3, C4, C5 Family

The C3 family proteins, C3, C4 and C5, are structurally homologues and their effect during complement activation is based on similar molecular mechanism. Upon cleavage by the convertase complexes these molecules undergo major conformational changes which enable them to bind the next components of the complement system (C3b: FB; C4b: C2; C5b: C6, C7) and attach to the surface. These are crucial events for organizing the complement activation. C3, C4 and C5 are members of the α_2_-macroglobulin family. They have evolved most likely from a common ancestral gene (98). C3 and C4, like α_2_-macroglobulin, contains the internal thioester bond, which is necessary to localize the complement activation to the surface of the target cell (99). All three proteins are synthesized as single chain pre-pro molecules and undergo posttranslational modifications in the endoplasmic reticulum and in the Golgi. Probably the most important posttranslational modification is the proteolytic cleavage by proprotein convertases, which yields the mature subunit structure.

**Figure 3:**
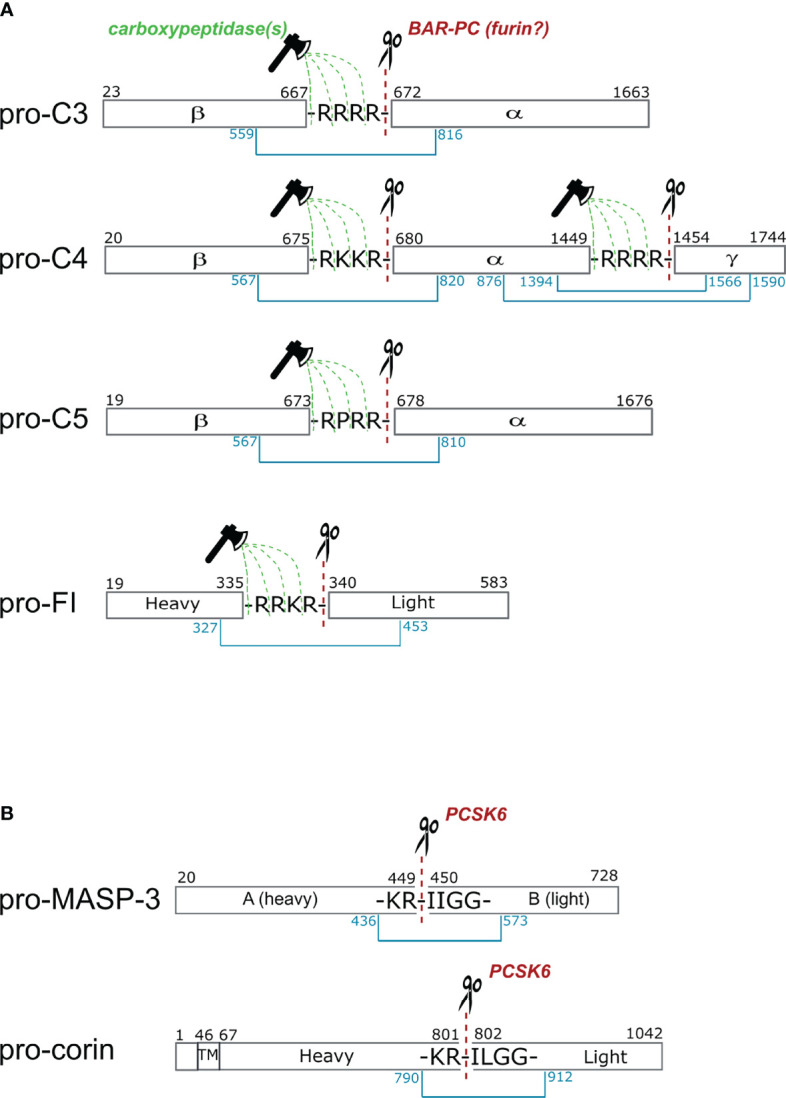
Cleavage sites of proprotein convertases after paired basic residues in C3, C4, C5, factor I, MASP-3 and corin. **(A)** Complement proteins that are processed intracellularly by proprotein convertases. Cleavage sites of basic amino acid residue-specific proprotein convertases (BAR-PC) are indicated as red dashed lines and scissors. Residual amino acids are trimmed off by carboxypeptidases marked with green dashed lines and ax. After BAR-PC cleavage, the complement proteins disintegrate into multiple chains: α and β chains in C3 and C5, α, β and γ chains in C4 and; heavy and light chains in the case of FI. The light chain of FI represents the serine protease domain in the active enzyme. Chains are held together by disulfide bonds indicated with blue lines. Black numbers show the first and last amino acids of each chain, while blue numbers stand for cysteines that form interchain disulfide bonds. **(B)** These two proteins are processed extracellularly by proprotein convertases. The sequences are very similar near the cleavage sites in MASP-3 and corin. The cleavage sites in the propeptide segment performed by PCSK6 are illustrated with red dashed lines and scissors. The with disulfide bonds connecting the heavy and light chains are indicated with blue lines. In corin the transmembrane (TM) region is also shown, while the activation takes place in the extracellular part of the protein.

C3 is mainly synthesized in the liver. The pre-pro molecule consists of 1663 amino acids. The α and β chains are separated by a tetra-arginine sequence between Ala^667^ and Ser^672^ (**Figure 3A**). In an experiment Misumi et al. showed that co-expression of C3 and furin in COS cells resulted in correctly processed C3 (100). Without furin co-expression only about half of the pro-C3 expressed was processed by endogenous proprotein convertase activity of the cells. This experiment proved that a proprotein convertase is responsible for the correct processing of pro-C3, but we cannot claim that furin is the exclusive processing enzyme. Very likely furin is the major processing protease, however, because of the redundant substrate specificity we cannot exclude the role of other proprotein convertases. As we mentioned in the previous section PACE4 also can process pro-C3 (78).

The primary site of C4 synthesis is also the liver. The pre-pro single-chain C4 consists of 1744 amino acids. In the polypeptide chain two tetrabasic sequences that separates the β-, α-, and γ-chains, that are ideal cleavage sequences for proprotein convertases. Between the β- and α-chains there is a Arg-Lys-Lys-Arg sequence and between the α- and γ-chains there is a tetra-arginine sequence (**Figure 3A**). Based on the analogy of C3, it is very probable that pro-C4 is processed by a furin-like proprotein convertase, but there is no direct experimental evidence for this. In 1980, before the discovery of the proprotein convertases, Goldberger and Colten showed that plasmin can process pro-C4 correctly *in vitro* (101). This is possible, since other proteases having similar substrate specificity can cleave at the tetrabasic sequence. Plasmin is a trypsin-like protease that cleaves after basic amino acids (Lys or Arg). After the discovery of the proprotein convertases (in the 1990s), however, it became evident, that these enzymes are responsible for the processing of the pro-proteins along the secretory pathway (102). Interestingly, C4 is further processed after secretion in the in the blood plasma (103). A 22-residue peptide from the C-terminus of the α-chain is removed by a metalloprotease having elastase-like specificity (104).

C5 is synthetized by the liver hepatocytes. The pro-form of C5 contains 1676 amino acids (105). There is an Arg-Pro-Arg-Arg linker sequence between the β- and α-chains (**Figure 3A**). The pro-C5 processing occurs during secretion. Until now the processing enzyme has not been identified experimentally but based on the linker sequence, we can assume that a furin-like proprotein convertase is responsible for the cleavage.

It is important to note, that furin, and the other proprotein convertases cleaves after the last amino acid of the tetrabasic linker sequence. After this cleavage the tetrabasic sequence remains attached to the C-terminus of one of the chains (β-chain of C3 and C5, and β- and α-chains of C4). In the mature proteins, however, the tetrabasic sequences are completely removed. This might be important for the full biological activity. We know from the example of insulin, that after proprotein convertase-mediated cleavage the C-terminally exposed basic residues are trimmed off by carboxypeptidases to generate fully active peptides (47, 54). One can assume, that in the case of C3, C4 and C5 a similar mechanism takes place, however the carboxypeptidase cleavage might occur in the blood plasma after secretion.

### Factor I

Factor I (FI) is a major regulator of complement activation since it is able to degrade activated C3b and C4b in the presence of its cofactors and dismantle C3b convertases of all pathways. The proper functioning of FI is crucial to protect host cells from unwanted complement attack and also to produce fragments for opsonization. FI is a glycoprotein which is found in its active two-chain form of 88 kDa in human blood. As the majority of complement serine proteases, FI is synthetized as pre-proprotein primarily in hepatocytes (106). Several other cell types also express functional FI such as monocytes, myoblasts, fibroblasts, human umbilical vein embryonic cells (HUVEC) and keratinocytes (107). FI is a multidomain protein of five domains namely FIMAC (factor I membrane attack complex) domain, CD5 domain (also called SRCR domain), two LDLRA (low-density lipoprotein receptor type A) domains located on the heavy chain, and SP (serine protease) domain on the light chain (108). Compared to other complement proteases, Factor I is different in terms of activation. It is the only complement protease that is activated inside the cell during secretion. After being released from the cell it circulates as an active protease without any known inhibitor in the blood. The physiological function of FI is controlled through cofactors which modify the affinity and specificity towards its substrates. Crystal structure indicated that the SP domain in free FI is partially distorted (26). This finding explains the low catalytic activity of FI towards small peptide substrates (109). However, the SP domain showed stabilized conformation in the ternary complex of FI with its cofactor FH and its substrate C3b. Data revealed that the SP domain can reach its fully active conformation only in complex with its substrates and cofactors (28).

The single polypeptide chain synthesized intracellularly contains a signal sequence and an activation linker (R^336^-R-K-R^339^). The precursor undergoes posttranslational modification while migrates through the Golgi, gets three sites glycosylated on the light and also on the heavy chain and afterwards the linker region is cut out. The sequence of the linker region is a typical BAR-PC recognition site (**Figure 3A**), nonetheless the exact activator of FI is still unknown. Although the activation of complement FI is not fully understood, several experimental facts indicate involvement of proprotein convertases in the process. Early attempts to produce active two-chain enzyme were carried out in different hepatoma derived cell lines (Hep3B, NPLC-KC and HepG2) (110). The proportion of the single-chain pro-FI and the mature protein in different cell cultures vary from each other. NPLC-KC and Hep3b cells predominantly produced the two-chain form while HepG2 cells mostly secreted the single-chain form of FI. The authors also showed that other multidomain complement protein such as C3, C4 and C5 were secreted as mature proteins by the mentioned three hepatoma cell lines. The major product did not differ regardless of their source. These findings indicate that the intracellular posttranslational modification of FI may require other activator than that of C3, C4 or C5. The possibility that cleavage of proprotein takes place extracellularly was ruled out since pro-FI was not activated when incubated in human plasma.

Active FI can also be expressed by insect cells. Baculovirus expression system using *Trichopulsia ni* (High Five) cells yielded fully processed FI, although molecular weight of the protease was somewhat different compared to the serum protein due to altered glycosylation (111). In order to improve the yield of active form, FI was co-expressed in mammalian cells with a possible activator, furin (112). Monkey kidney cells (COS-1) secreted more than 90% of FI as single-chain zymogen, while Chinese hamster ovary cells (CHO-K1) expressed 50% of that. However, when cells were co-transfected with both FI and furin cDNA, the ratio of two forms significantly changed. Processing became complete, portion of the mature FI moved above 90%. Human embryonic kidney cells (HEK293) co-transfected with furin resulted in mature FI sufficient quality and quantity for crystallization (28). Furin or other intracellular proprotein convertases could cleave the linker region between Arg^339^ and Ile^340^, however another enzymatic step is still needed. Residual basic amino acids on the new C-terminus have to be cut off otherwise FI could not reach full activity. A well-known example for this process is the maturation of insulin (54). Although not every detail of the intracellular posttranslational modifications of FI have been fully clarified yet, the evidences listed above strongly support the involvement of proprotein convertases.

### Corin

Corin is an essential enzyme for maintaining normal blood pressure (113). It is a trypsin-like serine protease that cleaves pro-atrial natriuretic peptide (pro-ANP) to generate an active hormone, ANP. ANP is a cardiac hormone playing an important role in regulating salt-water balance and blood pressure. The structure and function of corin resembles that of the complement proteases in several aspects. Corin is a multidomain serine protease. It is a type II transmembrane protease having an N-terminal transmembrane domain and a C-terminal trypsin-like serine protease domain. Between the transmembrane domain and the serine protease domain there are two frizzled domains, eight low density lipoprotein receptor repeats and a scavenger receptor domain. It is highly expressed in cardiomyocytes, and it is expected to exert its function on the surface of these cells (114). Recently, however, soluble corin was detected in the human circulation, indicating that corin is shed from the cardiomyocytes (115). Corin, like most of the complement proteases, is synthetized as an inactive one-chain zymogen, and it is activated by limited proteolysis at a conserved (**Figure 3B**) site between Arg^801^ and Ile^802^ (116). The resulting two-chain molecule shows full enzymatic activity. The two polypeptide chains are held together by a disulfide bond. Failure of corin activation can result in developing hypertension and heart disease. Accordingly, naturally occurring corin gene variants with impaired zymogen activation have been identified in patients (117). The activation sequence and the mode of activation of corin resembles to that of the plasma cascade enzymes. Corin has no autoactivation capacity therefore it must be activated by other protease. Since the trypsin-like enzymes having a common evolutionary origin form a protease network in the blood plasma and on the surface of different cells (e.g. endothelial cells, leucocytes, cardiomyocytes, etc.), it seemed obvious that the activator protease should be sought among them. It was a big surprise, however, when it was discovered, that the activator protease is a proprotein convertase, PCSK6 (aka PACE4) (55). A proprotein convertase-specific inhibitor completely blocked the activation of corin, and a similar effect was exerted by using siRNAs against PCSK6 expression. PCSK6 knockout mice have only zymogen corin in the heart and they show symptoms of hypertension. The first reason for the surprise was that the proprotein convertases represent a different evolutionary line of the serine proteases (the subtilisins) compared to the trypsin-like proteases (118). It was the first example in the blood for a cross-talk between the proprotein convertase and the trypsin-like protease network. The second reason for the surprise was that corin is secreted as a zymogen and the proprotein convertase-mediated activation takes place on the surface of the cells. The proprotein convertases usually act inside the cells, along the secretory pathway, as we have seen in the example of C3, C4, C5 and FI. In the case of corin, however, it was shown that corin and PCSK6 use different secretory pathways. Chen et al. demonstrated that a soluble corin mutant lacking the transmembrane domain was activated by PCSK6 in the conditioned medium of HEK293 cells but not intracellularly (119). Blocking PCSK6 secretion by monensin and subsequent immunostaining indicated that corin and PCSK6 were secreted *via* different intracellular pathways, which may explain the lack of corin activation inside the cell. The detection of secreted PCSK6 in the human blood indicated that PCSK6 may process other proteins in the circulation thereby modulating the effect of the proteolytic cascade systems (56).

### MASP-3

MASP-3 was discovered as the third serine protease component of the lectin pathway of complement (120). Originally it had been thought to negatively regulate the lectin pathway, as it had no known natural substrate despite having a functional serine protease domain (120). MASP-3 had been implicated to cleave insulin-like growth factor-binding protein 5 (IGFBP-5), however, *in vivo* relevance of this reaction has not been established (121). More importantly, certain mutations that effect the catalytic activity of MASP-3 can cause a serious developmental disorder, the 3MC (Malpuech-Michels-Mingarelli-Carnevale) syndrome (122), which is characterized by cognitive defects, craniofacial dismorphysm, and other abnormalities. It is interesting that PCSK6 KO mice (58, 123) and 3MC (human) patients (124) show similar craniofacial deformities. The exact pathomechanism of this disease remains to be solved, however, it indicates that MASP-3 might have a role outside the regular scheme of the complement system, and both MASP-3 and PCSK6 may be involved in the same mechanistic route.

MASP-3 is expressed as a splice variant from the *MASP1* gene, which has three products that have been identified at the protein level, MASP-1, MASP-3 and MAp44 (125–127). Map44 is indeed a regulatory molecule without any catalytic activity. MASP-1 and MASP-3 are, however, functional serine proteases. The first evidence that MASP-1 and/or MASP-3 might play a role in the activation of the alternative pathway was provided by the group of Teizo Fujita (128). They generated *MASP1* knockout mice by replacing the second exon encoding a common region shared by both proteases (129). Serum derived from mice lacking both proteases had minimal alternative pathway activity, because it contained proenzymic factor D (pro-FD). Initially MASP-1 was implicated as the pro-FD activator (maturase) (128). While *in vitro*, MASP-1, and a number of other proteases, can process pro-FD in to FD (130), *in vivo* MASP-3 is the FD maturase enzyme (24, 131, 132). Under normal resting conditions, unperturbed by ongoing coagulation or complement activation, MASP-3 is already present predominantly in the activated, two-chain form (23), while MASP-1 and other implicated proteases are proenzymic. Hence, in normal human individuals MASP-3 has a homeostatic role: it is present (mostly) as an active enzyme and continuously activates pro-FD, which is, as a result, also present predominantly in the mature FD form (24, 133). It worth mentioning that human and mouse MASP-3 seems to behave very similarly in this respect therefore the FD maturase function of MASP-3 is probably conserved among mammalian species (132, 134).

As MASP-3 is mostly present in the blood as a mature, active enzyme the question immediately arises: how MASP-3 is activated? This question implies two problems, where does MASP-3 activation take place, and which protease or proteases activate MASP-3?

Notably, when MASP-3 was expressed in eukaryotic cell lines, insect or mammalian (CHO) cells, it was always secreted into the medium a one-chain zymogen (135–137). This strongly suggests that MASP-3 is not activated intracellularly. For the first time, MASP-3 was expressed in insect cells, and it was convincingly proved that MASP-3 cannot autoactivate (135). Interestingly, when isolated MASP-3 was stored at 4 °C for months, it slowly became activated by an unknown contaminating protease. The cleavage occurred even if the catalytically inactive S664A (precursor numbering) mutant MASP-3 was isolated and then stored at 4 °C. The activation did not occur at 37 °C suggesting that the activating enzyme is heat-labile. Although this insect protease has never been identified, presumably it was a BAR-PC, which seems to be also secreted like PACE4 (PCSK6).

**Figure 4:**
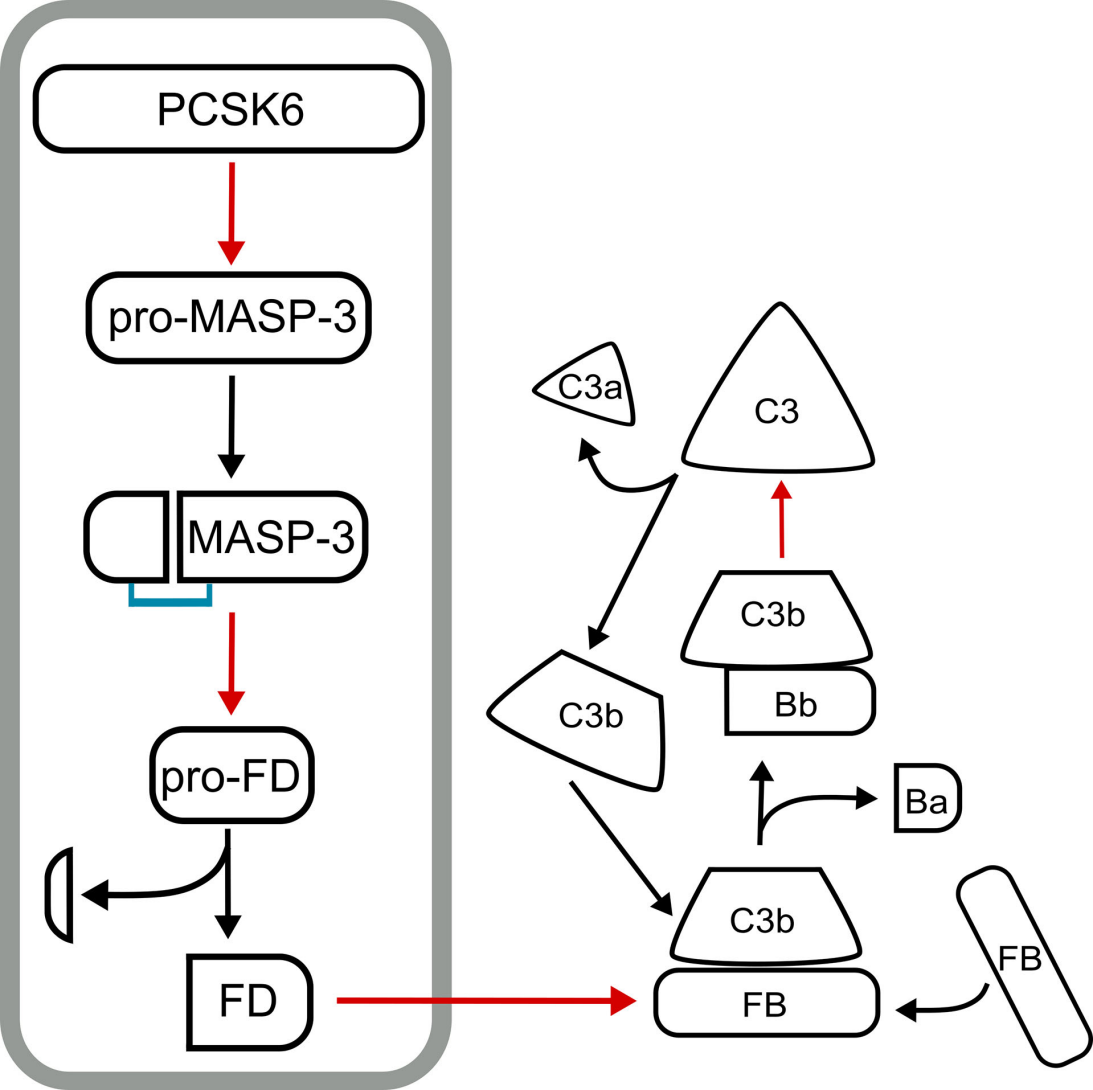
The PCSK6-MASP-3-FD axis. The proenzyme form of MASP-3 (pro-MASP-3) is constitutively activated in the blood by PCSK6 (PACE4). Active MASP-3 cleaves pro-factor D (pro-FD) also constitutively. These two cleavage events are required to keep the alternative pathway of complement ready for action on appropriate surfaces. The PCSK6-MASP-3-FD axis is in a grey box, while subsequent events of the alternative pathway following FD activation are shown on the right. Red arrows point from a protease to its substrate, while black arrows indicate conversion.

We have recently identified PCSK6 (PACE4) as the most likely activator of MASP-3 and showed that the activation of MASP-3 takes place in the blood using human samples (138). Evidences that support this mechanism are as follows. The inactive S664A MASP-3 variant became cleaved (“activated”) when added to blood samples containing Ca^2+^ (e.g. hirudin plasma, or serum). On the other hand, the reaction did not occur in EDTA plasma. It important to note again that proprotein convertases require Ca^2+^ for their activity. Most importantly, the “activation” (cleavage) reaction of MASP-3 S664A was completely blocked in hirudin plasma by the general proprotein convertase inhibitor decanoyl-Arg-Val-Lys-Arg-chloromethylketone (dec-RVKR-cmk) specific for BAR-PCs. Hence we were looking for a proprotein convertase, which is secreted and cleaves after paired basic residues. It is remarkable that the protease, corin, which is also activated in the blood by PCSK6 (PACE4), has a very similar sequence motif around the activation site to that of MASP-3 (**Figure 3B**). Nevertheless, there is another BAR-PC, which is secreted, namely the PC5A, a major isoform of PCSK5. We have expressed both PCSK6 (PACE4) and PCSK5 (PC5A isoform) in CHO cells, and found that both could activate MASP-3 *in vitro*. We have also shown that the MASP-3 activator in hirudin plasma is also heat-labile, because the activator remained active for a longer period of time at 25 °C. It was published that PACE4 (PCSK6) is very sensitive to even moderately high temperatures such as 37 °C (61). And finally, we and others have shown that PCSK6 is present in the blood as a soluble protein (55, 56), whereas PCSK5 (PC5A) is not, or at least no one could detect it. Taken together, PCSK6 (PACE4) emerges as possibly the sole, or at least the major activator of MASP-3 in human blood. As MASP-3 is the activator pro-FD, and FD is essential for the alternative pathway of complement, we have identified PCSK6 as an essential, highest level activator of complement. It is important to note that this activation sequence occurs constantly in normal healthy individuals in a homeostatic fashion. The described mechanism is summarized on **Figure 4**.

## Conclusion And Perspectives

**Figure 5:**
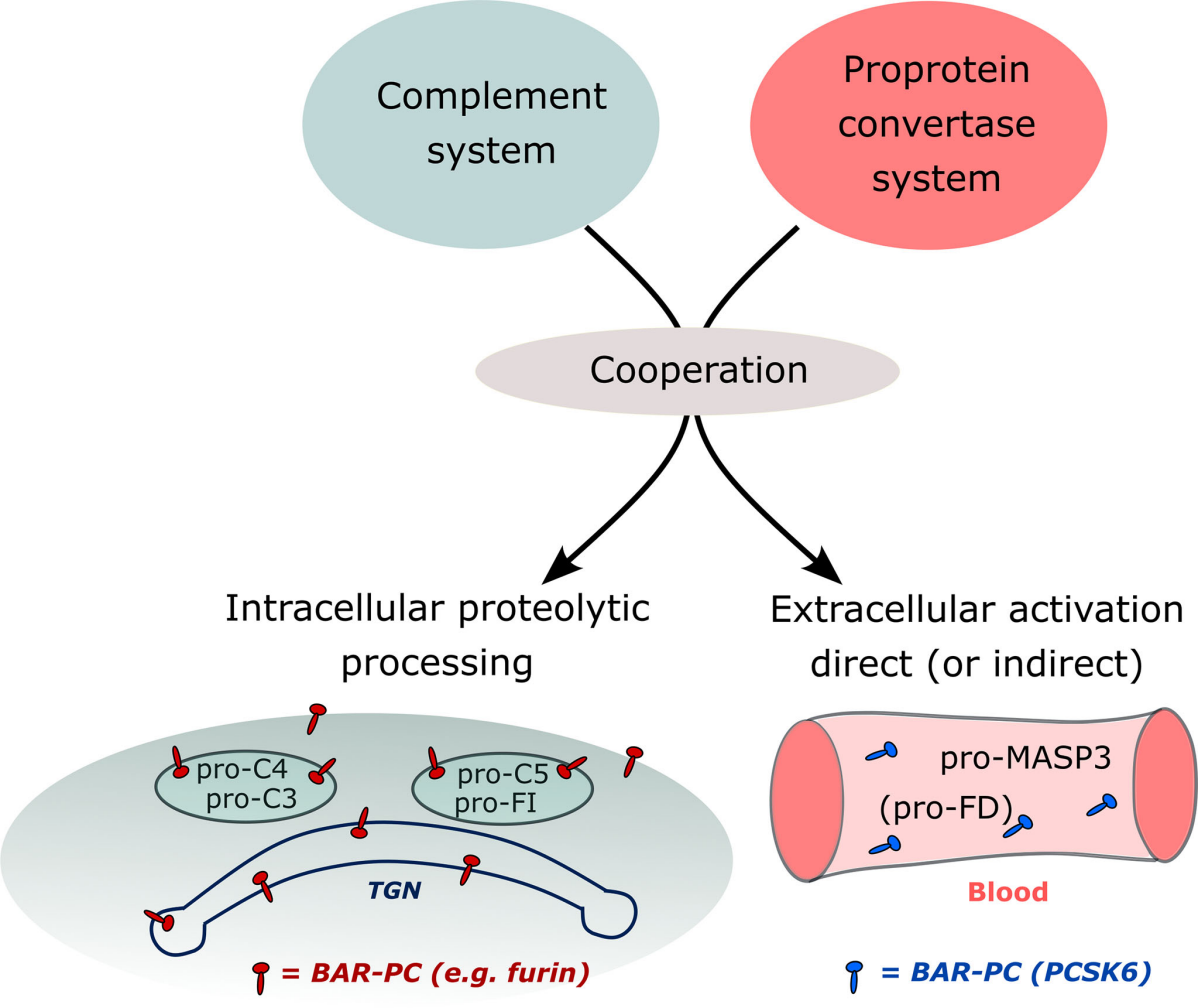
Processing of complement proteins by proprotein convertases in different compartments of the body. Cooperation between the complement system and BAR-PCs (basic amino acid residue-specific proprotein convertases) results in proteolytic processing of complement proteins inside the cell or extracellularly in the blood. BAR-PCs such as furin localize to the membrane of the trans-Golgi network (TGN), secretory vesicles and endosomes. Processing of pro-C3, pro-C4, pro-C5, and pro-factor I probably occurs in these compartments. The direct activation of pro-MASP-3 occurs in the blood by circulating PCSK6 (PACE4). Pro-factor D is the substrate of active MASP-3 therefore it is an example of indirect activation by BAR-PCs.

The complement system is a part of the proteolytic network of the human body. It has been known for a long time that the blood cascade systems – complement, coagulation, fibrinolysis – interact with each other; there are cross-activation steps, and there are common inhibitors. For example, MASP-1 can initiate coagulation by activating prothrombin (139); and C1-inhibitor, the major inhibitor of the classical and the lectin pathway, also inhibits the contact activation system (FXIIa and plasma kallikrein). It was also described that zymogen activation can occur between proteases of different mechanistic groups. Plasmin can initiate the activation of several metalloprotease zymogens (e.g. MMP-1/-3/-9/-13). In this way plasmin could facilitate matrix degradation during various physiological and pathological events (140). Another example of cross-class activation is when the serine protease granzymes, released by cytotoxic T cells or natural killer cells, initiate the apoptosis by activating the cysteine protease caspases in the target cells (141). The discovery that a proprotein convertase is involved in the alternative pathway activation drew attention to the cooperation between the proprotein convertases and the complement system (**Figure 5**). Although both the proprotein convertases and the complement proteases are serine proteases, they represent quite different evolutionary lines (i.e. trypsin-like vs. subtilisin-like proteases).

PCSK6 is the highest level initiator enzyme of the alternative pathway. In the pre-initiation phase of the alternative pathway, before any activation signal appears, PCSK6 activates MASP-3, which in turn activates FD (**Figure 4**). These reactions ensure that when C3b appears on the surface, the alternative pathway can be started immediately with high efficiency because sufficient amount of active FD is available. The proprotein convertases also play a crucial role in the intracellular processing of several complement proteins such as FI, C3, C4 and C5 (**Figure 5**). Knowing these interactions, we cannot rule out that there are other hidden connections between the proprotein convertases and the complement system.

For example, the anaphylatoxins C3a and C5a can modulate the tumor microenvironment by promoting tumorigenesis through the recruitment of myeloid-derived suppressor cells for inhibiting antitumor CD8+ T cells and through induction of neovascularization (142). Complement components are present in the tumor: they can be secreted either by the tumor cells, or by tumor-infiltrating immune cells, or they can enter the tumor through the vasculature. Cancer cell-born proteases can directly cleave C3 and C5 generating the anaphylatoxins. Nitta et al. demonstrated that cancer cells display a serine protease, which cleaves C5 and releases C5a (143). This protease is likely a proprotein convertase since its activity is completely blocked by the BAR-PC-specific inhibitor (dec-RVKR-cmk).

Also, proprotein convertases may contribute to the intracellular action of the complement proteins, since the anaphylatoxins can be released intracellularly (144, 145). Further studies are needed to reveal all the functional connections between the components of the complex protease network of the immune system including the proprotein convertases and the complement proteases.

## Author Contributions

All authors listed have made a substantial, direct, and intellectual contribution to the work and approved it for publication.

## Funding

The study was supported by the Eötvös Loránd Research Network (ELKH) grant KEP-5/2021, and the National Research, Development and Innovation Office (NKFIH) grants K134711 and 2020-1.1.2-PIACI-KFI-2021-00273.

## Conflict of Interest

The authors declare that the research was conducted in the absence of any commercial or financial relationships that could be construed as a potential conflict of interest.

## Publisher’s Note

All claims expressed in this article are solely those of the authors and do not necessarily represent those of their affiliated organizations, or those of the publisher, the editors and the reviewers. Any product that may be evaluated in this article, or claim that may be made by its manufacturer, is not guaranteed or endorsed by the publisher.

## Abbreviations

↓, indicates a cleavage site; 3MC, Malpuech-Michels-Mingarelli-Carnevale (syndrome) aka, also known as; BAR-PC, basic amino acid residue-specific proprotein convertase; C1…C9, complement component 1-9; C1q, C1r, C1s, subcomponents of C1; C4BP, C4b-binding protein; CHO, Chinese hamster ovary (cells); CHRD, cysteine- and histidine-rich domain; CR1, complement receptor 1 (aka C3b/C4b receptor); CRD, cysteine-rich domain; dec-RVKR-cmk, decanoyl-Arg-Val-Lys-Arg-chloromethylketone; ER, endoplasmic reticulum; FB, factor B; FD, factor D; FH, factor H; FI, factor I; FIMAC, factor I membrane attack complex (domain); Gdf11, growth/differentiation factor 11; IGF-II, insulin-like grow factor-II; IGFBP-5, insulin-like growth factor-binding protein 5; HEK, human embryonic kidney (cells); Hep3B, a human hepatoma cell line; HepG2, a human hepatoma cell line; HDL, high-density lipoprotein; HSPG, heparan sulfate proteoglycans; HUVEC, human umbilical vein endothelial cells; L1, neural adhesion molecule L1; LDL, low-density lipoprotein; LDLRA, low-density lipoprotein receptor type A (domain); LDLR, low-density lipoprotein receptor; MASP, mannan-binding lectin-associated serine protease; MAp44, mannan-binding lectin-associated protein 44 (kDa); MBL, mannan-binding lectin; MBTPS1, membrane-bound transcription factor protease, site-1 (aka SKI-1); MCP, membrane cofactor protein; MMP, matrix metalloprotease; NARC-1, neural apoptosis regulated convertase 1; NEC, neuroendocrine convertase; NPLC-KC, a human hepatoma cell line; PACE, paired basic amino acid-cleaving enzyme (aka furin, PCSK3); PACE4, paired basic amino acid-cleaving enzyme 4 (aka PCSK6); PC, proprotein convertase; PCSK, proprotein convertase subtilisin/kexin; PCSK1-9, proprotein convertase subtilisin/kexin type 1-9; PC5A, secreted, short isoform of PCSK5 (aka PC6A); PC5B, membrane-bound, long isoform of PCSK5 (aka PC6B); PDGF, platelet-derived growth factor; RGD, tripeptide motif of Arg-Gly-Asp; RPTPμ, receptor protein tyrosine phosphatase μ; S1P, site-1 protease; SKI-1, subtilisin/kexin-isozyme 1 (aka MBTPS1); SP, serine protease (domain); SPC4, subtilisin-like proprotein convertase 4 (aka PCSK6); TGN, trans-Golgi network; TGF-β, transforming growth factor-β; TIMP, tissue inhibitors of metalloproteases; VSMC, vascular smooth muscle cells.

## References

[1] Merle NS Church SE Fremeaux-Bacchi V Roumenina LT. Complement System Part I - Molecular Mechanisms of Activation and Regulation. Front Immunol (2015) 6:262. doi: 10.3389/fimmu.2015.00262 26082779 PMC4451739

[2] Merle NS Noe R Halbwachs-Mecarelli L Fremeaux-Bacchi V Roumenina LT. Complement System Part II: Role in Immunity. Front Immunol (2015) 6:257. doi: 10.3389/fimmu.2015.00257 26074922 PMC4443744

[3] Hajishengallis G Reis ES Mastellos DC Ricklin D Lambris JD. Novel Mechanisms and Functions of Complement. Nat Immunol (2017) 18:1288–98. doi: 10.1038/ni.3858 PMC5706779 29144501

[4] Ricklin D Hajishengallis G Yang K Lambris JD. Complement: A Key System for Immune Surveillance and Homeostasis. Nat Immunol (2010) 11:785–97. doi: 10.1038/ni.1923 PMC2924908 20720586

[5] Sim RB Tsiftsoglou SA. Proteases of the Complement System. Biochem Soc Trans (2004) 32:21–7. doi: 10.1042/bst0320021 14748705

[6] Morgan BP Harris CL. Complement, a Target for Therapy in Inflammatory and Degenerative Diseases. Nat Rev Drug Discovery (2015) 14:857–77. doi: 10.1038/nrd4657 PMC7098197 26493766

[7] Mastellos DC Ricklin D Lambris JD. Clinical Promise of Next-Generation Complement Therapeutics. Nat Rev Drug Discovery (2019) 18:707–29. doi: 10.1038/s41573-019-0031-6 PMC7340853 31324874

[8] Dobó J Kocsis A Gál P. Be on Target: Strategies of Targeting Alternative and Lectin Pathway Components in Complement-Mediated Diseases. Front Immunol (2018) 9:1851. doi: 10.3389/fimmu.2018.01851 30135690 PMC6092519

[9] Diebolder CA Beurskens FJ de Jong RN Koning RI Strumane K Lindorfer MA. Complement is Activated by IgG Hexamers Assembled at the Cell Surface. Science (2014) 343:1260–3. doi: 10.1126/science.1248943 PMC4250092 24626930

[10] Sharp TH Boyle AL Diebolder CA Kros A Koster AJ Gros P. Insights Into IgM-Mediated Complement Activation Based on *in Situ* Structures of IgM-C1-C4b. Proc Natl Acad Sci U S A (2019) 116:11900–5. doi: 10.1073/pnas.1901841116 PMC6575175 31147461

[11] Zwarthoff SA Widmer K Kuipers A Strasser J Ruyken M Aerts PC. C1q Binding to Surface-Bound IgG is Stabilized by C1r2s2 Proteases. Proc Natl Acad Sci U S A (2021) 118:e2102787118. doi: 10.1073/pnas.2102787118 34155115 PMC8256010

[12] Holmskov U Thiel S Jensenius JC. Collections and Ficolins: Humoral Lectins of the Innate Immune Defense. Annu Rev Immunol (2003) 21:547–78. doi: 10.1146/annurev.immunol.21.120601.140954 12524383

[13] Héja D Kocsis A Dobó J Szilágyi K Szász R Závodszky P. Revised Mechanism of Complement Lectin-Pathway Activation Revealing the Role of Serine Protease MASP-1 as the Exclusive Activator of MASP-2. Proc Natl Acad Sci U S A (2012) 109:10498–503. doi: 10.1073/pnas.1202588109 PMC3387078 22691502

[14] Lachmann PJ. Looking Back on the Alternative Complement Pathway. Immunobiology (2018) 223:519–23. doi: 10.1016/j.imbio.2018.02.001 29525356

[15] Harboe M Ulvund G Vien L Fung M Mollnes TE. The Quantitative Role of Alternative Pathway Amplification in Classical Pathway Induced Terminal Complement Activation. Clin Exp Immunol (2004) 138:439–46. doi: 10.1111/j.1365-2249.2004.02627.x PMC1809239 15544620

[16] Pangburn MK Schreiber RD Müller-Eberhard HJ. Formation of the Initial C3 Convertase of the Alternative Complement Pathway. Acquisition of C3b-Like Activities by Spontaneous Hydrolysis of the Putative Thioester in Native C3. J Exp Med (1981) 154:856–67. doi: 10.1084/jem.154.3.856 PMC2186450 6912277

[17] Mannes M Dopler A Zolk O Lang SJ Halbgebauer R Höchsmann B. Complement Inhibition at the Level of C3 or C5: Mechanistic Reasons for Ongoing Terminal Pathway Activity. Blood (2021) 137:443–55. doi: 10.1182/blood.2020005959 33507296

[18] Roumenina LT. Terminal Complement Without C5 Convertase? Blood (2021) 137:431–2. doi: 10.1182/blood.2020010133 33507301

[19] Tegla CA Cudrici C Patel S Trippe R Rus V Niculescu F. Membrane Attack by Complement: The Assembly and Biology of Terminal Complement Complexes. Immunol Res (2011) 51:45–60. doi: 10.1007/s12026-011-8239-5 21850539 PMC3732183

[20] Krem MM Di Cera E. Evolution of Enzyme Cascades From Embryonic Development to Blood Coagulation. Trends Biochem Sci (2002) 27:67–74. doi: 10.1016/s0968-0004(01)02007-2 11852243

[21] Gál P Harmat V Kocsis A Bián T Barna L Ambrus G. A True Autoactivating Enzyme. Structural Insight Into Mannose-Binding Lectin-Associated Serine Protease-2 Activations. J Biol Chem (2005) 280:33435–44. doi: 10.1074/jbc.M506051200 16040602

[22] Megyeri M Harmat V Major B Végh Á Balczer J Héja D. Quantitative Characterization of the Activation Steps of Mannan-Binding Lectin (MBL)-Associated Serine Proteases (MASPs) Points to the Central Role of MASP-1 in the Initiation of the Complement Lectin Pathway. J Biol Chem (2013) 288:8922–34. doi: 10.1074/jbc.M112.446500 PMC3610966 23386610

[23] Oroszlán G Dani R Szilágyi A Závodszky P Thiel S Gál P. Extensive Basal Level Activation of Complement Mannose-Binding Lectin-Associated Serine Protease-3: Kinetic Modeling of Lectin Pathway Activation Provides Possible Mechanism. Front Immunol (2017) 8:1821. doi: 10.3389/fimmu.2017.01821 29326707 PMC5741598

[24] Pihl R Jensen L Hansen AG Thøgersen IB Andres S Dagnæs-Hansen F. Analysis of Factor D Isoforms in Malpuech-Michels-Mingarelli-Carnevale Patients Highlights the Role of MASP-3 as a Maturase in the Alternative Pathway of Complement. J Immunol Baltim Md 1950 (2017), 199:2158-2170 ji1700518. doi: 10.4049/jimmunol.1700518 28794230

[25] Jing H Macon KJ Moore D DeLucas LJ Volanakis JE Narayana SV. Structural Basis of Profactor D Activation: From a Highly Flexible Zymogen to a Novel Self-Inhibited Serine Protease, Complement Factor D. EMBO J (1999) 18:804–14. doi: 10.1093/emboj/18.4.804 PMC1171173 10022823

[26] Roversi P Johnson S Caesar JJE McLean F Leath KJ Tsiftsoglou SA. Structural Basis for Complement Factor I Control and its Disease-Associated Sequence Polymorphisms. Proc Natl Acad Sci U S A (2011) 108:12839–44. doi: 10.1073/pnas.1102167108 PMC3150940 21768352

[27] Forneris F Ricklin D Wu J Tzekou A Wallace RS Lambris JD. Structures of C3b in Complex With Factors B and D Give Insight Into Complement Convertase Formation. Science (2010) 330:1816–20. doi: 10.1126/science.1195821 PMC3087196 21205667

[28] Xue X Wu J Ricklin D Forneris F Di Crescenzio P Schmidt CQ. Regulator-Dependent Mechanisms of C3b Processing by Factor I Allow Differentiation of Immune Responses. Nat Struct Mol Biol (2017) 24:643–51. doi: 10.1038/nsmb.3427 PMC5773341 28671664

[29] Rooryck C Diaz-Font A Osborn DPS Chabchoub E Hernandez-Hernandez V Shamseldin H. Mutations in Lectin Complement Pathway Genes COLEC11 and MASP1 Cause 3MC Syndrome. Nat Genet (2011) 43:197–203. doi: 10.1038/ng.757 21258343 PMC3045628

[30] Yongqing T Wilmann PG Reeve SB Coetzer TH Smith AI Whisstock JC. The X-Ray Crystal Structure of Mannose-Binding Lectin-Associated Serine Proteinase-3 Reveals the Structural Basis for Enzyme Inactivity Associated With the Carnevale, Mingarelli, Malpuech, and Michels (3MC) Syndrome. J Biol Chem (2013) 288:22399–407. doi: 10.1074/jbc.M113.483875 PMC3829330 23792966

[31] Huntington JA. Serpin Structure, Function and Dysfunction. J Thromb Haemost JTH (2011) 9 Suppl 1:26–34. doi: 10.1111/j.1538-7836.2011.04360.x 21781239

[32] Gettins PGW. Serpin Structure, Mechanism, and Function. Chem Rev (2002) 102:4751–804. doi: 10.1021/cr010170+ 12475206

[33] Davis AE Lu F Mejia P. C1 Inhibitor, a Multi-Functional Serine Protease Inhibitor. Thromb Haemost (2010) 104:886–93. doi: 10.1160/TH10-01-0073 20806108

[34] Paréj K Dobó J Závodszky P Gál P. The Control of the Complement Lectin Pathway Activation Revisited: Both C1-Inhibitor and Antithrombin are Likely Physiological Inhibitors, While α2-Macroglobulin is Not. Mol Immunol (2013) 54:415–22. doi: 10.1016/j.molimm.2013.01.009 23399388

[35] Forneris F Wu J Gros P. The Modular Serine Proteases of the Complement Cascade. Curr Opin Struct Biol (2012) 22:333–41. doi: 10.1016/j.sbi.2012.04.001 22560446

[36] van den Bos RM Pearce NM Granneman J Brondijk THC Gros P. Insights Into Enhanced Complement Activation by Structures of Properdin and Its Complex With the C-Terminal Domain of C3b. Front Immunol (2019) 10:2097. doi: 10.3389/fimmu.2019.02097 31552043 PMC6736995

[37] Pedersen DV Gadeberg TAF Thomas C Wang Y Joram N Jensen RK. Structural Basis for Properdin Oligomerization and Convertase Stimulation in the Human Complement System. Front Immunol (2019) 10:2007. doi: 10.3389/fimmu.2019.02007 31507604 PMC6713926

[38] Gadjeva M Dodds AW Taniguchi-Sidle A Willis AC Isenman DE Law SK. The Covalent Binding Reaction of Complement Component C3. J Immunol Baltim Md 1950 (1998) 161:985–90. 9670979

[39] Sheehan M Morris CA Pussell BA Charlesworth JA. Complement Inhibition by Human Vitronectin Involves non-Heparin Binding Domains. Clin Exp Immunol (1995) 101:136–41. doi: 10.1111/j.1365-2249.1995.tb02289.x PMC1553293 7542572

[40] McDonald JF Nelsestuen GL. Potent Inhibition of Terminal Complement Assembly by Clusterin: Characterization of its Impact on C9 Polymerization. Biochemistry (1997) 36:7464–73. doi: 10.1021/bi962895r 9200695

[41] White RT Damm D Hancock N Rosen BS Lowell BB Usher P. Human Adipsin is Identical to Complement Factor D and is Expressed at High Levels in Adipose Tissue. J Biol Chem (1992) 267:9210–3. doi: 10.1016/S0021-9258(19)50409-4 1374388

[42] Seyfarth J Garred P Madsen HO. Extra-Hepatic Transcription of the Human Mannose-Binding Lectin Gene (Mbl2) and the MBL-Associated Serine Protease 1-3 Genes. Mol Immunol (2006) 43:962–71. doi: 10.1016/j.molimm.2005.06.033 16112196

[43] Armento A Ueffing M Clark SJ. The Complement System in Age-Related Macular Degeneration. Cell Mol Life Sci CMLS (2021) 78:4487–505. doi: 10.1007/s00018-021-03796-9 PMC8195907 33751148

[44] Zipfel PF Wiech T Gröne H-J Skerka C. Complement Catalyzing Glomerular Diseases. Cell Tissue Res (2021) 385:355–70. doi: 10.1007/s00441-021-03485-w PMC8523427 34613485

[45] Steiner DF. On the Discovery of Precursor Processing. Methods Mol Biol Clifton NJ (2011) 768:3–11. doi: 10.1007/978-1-61779-204-5_1 21805235

[46] Seidah NG. The Proprotein Convertases, 20 Years Later. Methods Mol Biol Clifton NJ (2011) 768:23–57. doi: 10.1007/978-1-61779-204-5_3 21805237

[47] Zhou A Webb G Zhu X Steiner DF. Proteolytic Processing in the Secretory Pathway. J Biol Chem (1999) 274:20745–8. doi: 10.1074/jbc.274.30.20745 10409610

[48] Thomas G. Furin at the Cutting Edge: From Protein Traffic to Embryogenesis and Disease. Nat Rev Mol Cell Biol (2002) 3:753–66. doi: 10.1038/nrm934 PMC1964754 12360192

[49] Seidah NG Prat A. The Biology and Therapeutic Targeting of the Proprotein Convertases. Nat Rev Drug Discovery (2012) 11:367–83. doi: 10.1038/nrd3699 22679642

[50] Garten W. Characterization of Proprotein Convertases and Their Involvement in Virus Propagation. Act Viruses Host Proteases (2018), 16:205–48. doi: 10.1007/978-3-319-75474-1_9

[51] Steiner DF Cunningham D Spigelman L Aten B. Insulin Biosynthesis: Evidence for a Precursor. Science (1967) 157:697–700. doi: 10.1126/science.157.3789.697 4291105

[52] Molloy SS Thomas L VanSlyke JK Stenberg PE Thomas G. Intracellular Trafficking and Activation of the Furin Proprotein Convertase: Localization to the TGN and Recycling From the Cell Surface. EMBO J (1994) 13:18–33. doi: 10.1002/j.1460-2075.1994.tb06231.x 7508380 PMC394775

[53] Seidah NG Chrétien M. Proprotein and Prohormone Convertases: A Family of Subtilases Generating Diverse Bioactive Polypeptides. Brain Res (1999) 848:45–62. doi: 10.1016/s0006-8993(99)01909-5 10701998

[54] Seidah NG Sadr MS Chrétien M Mbikay M. The Multifaceted Proprotein Convertases: Their Unique, Redundant, Complementary, and Opposite Functions. J Biol Chem (2013) 288:21473–81. doi: 10.1074/jbc.R113.481549 PMC3724608 23775089

[55] Chen S Cao P Dong N Peng J Zhang C Wang H. PCSK6-Mediated Corin Activation is Essential for Normal Blood Pressure. Nat Med (2015) 21:1048–53. doi: 10.1038/nm.3920 PMC4710517 26259032

[56] Yang S-F Chou R-H Lin S-J Li S-Y Huang P-H. Serum PCSK6 and Corin Levels are Not Associated With Cardiovascular Outcomes in Patients Undergoing Coronary Angiography. PLoS One (2019) 14:e0226129. doi: 10.1371/journal.pone.0226129 31825978 PMC6905542

[57] Kiefer MC Tucker JE Joh R Landsberg KE Saltman D Barr PJ. Identification of a Second Human Subtilisin-Like Protease Gene in the Fes/Fps Region of Chromosome 15. DNA Cell Biol (1991) 10:757–69. doi: 10.1089/dna.1991.10.757 1741956

[58] Constam DB Robertson EJ. SPC4/PACE4 Regulates a TGFbeta Signaling Network During Axis Formation. Genes Dev (2000) 14:1146–55. doi: 10.1101/gad.14.9.1146 PMC316583 10809672

[59] Benjannet S Savaria D Laslop A Munzer JS Chrétien M Marcinkiewicz M. Alpha1-Antitrypsin Portland Inhibits Processing of Precursors Mediated by Proprotein Convertases Primarily Within the Constitutive Secretory Pathway. J Biol Chem (1997) 272:26210–8. doi: 10.1074/jbc.272.42.26210 9334189

[60] Gordon VM Rehemtulla A Leppla SH. A Role for PACE4 in the Proteolytic Activation of Anthrax Toxin Protective Antigen. Infect Immun (1997) 65:3370–5. doi: 10.1128/iai.65.8.3370-3375.1997 PMC175476 9234799

[61] Sucic JF Moehring JM Inocencio NM Luchini JW Moehring TJ. Endoprotease PACE4 is Ca2+-Dependent and Temperature-Sensitive and can Partly Rescue the Phenotype of a Furin-Deficient Cell Strain. Biochem J (1999) 339(Pt 3):639–47. doi: 10.1042/bj3390639 PMC1220200 10215603

[62] Nagahama M Taniguchi T Hashimoto E Imamaki A Mori K Tsuji A. Biosynthetic Processing and Quaternary Interactions of Proprotein Convertase SPC4 (Pace4). FEBS Lett (1998) 434:155–9. doi: 10.1016/s0014-5793(98)00970-3 9738469

[63] Mayer G Hamelin J Asselin M-C Pasquato A Marcinkiewicz E Tang M. The Regulated Cell Surface Zymogen Activation of the Proprotein Convertase PC5A Directs the Processing of its Secretory Substrates. J Biol Chem (2008) 283:2373–84. doi: 10.1074/jbc.M708763200 18039650

[64] Shinde U Inouye M. Propeptide-Mediated Folding in Subtilisin: The Intramolecular Chaperone Concept. Adv Exp Med Biol (1996) 379:147–54. doi: 10.1007/978-1-4613-0319-0_16 8796319

[65] Dillon SL Williamson DM Elferich J Radler D Joshi R Thomas G. Propeptides are Sufficient to Regulate Organelle-Specific pH-Dependent Activation of Furin and Proprotein Convertase 1/3. J Mol Biol (2012) 423:47–62. doi: 10.1016/j.jmb.2012.06.023 22743102 PMC3444655

[66] Lipkind GM Zhou A Steiner DF. A Model for the Structure of the P Domains in the Subtilisin-Like Prohormone Convertases. Proc Natl Acad Sci U S A (1998) 95:7310–5. doi: 10.1073/pnas.95.13.7310 PMC22600 9636145

[67] Rovère C Luis J Lissitzky JC Basak A Marvaldi J Chrétien M. The RGD Motif and the C-Terminal Segment of Proprotein Convertase 1 are Critical for its Cellular Trafficking But Not for its Intracellular Binding to Integrin Alpha5beta1. J Biol Chem (1999) 274:12461–7. doi: 10.1074/jbc.274.18.12461 10212221

[68] Tsuji A Sakurai K Kiyokage E Yamazaki T Koide S Toida K. Secretory Proprotein Convertases PACE4 and PC6A are Heparin-Binding Proteins Which are Localized in the Extracellular Matrix. Potential Role of PACE4 in the Activation of Proproteins in the Extracellular Matrix. Biochim Biophys Acta (2003) 1645:95–104. doi: 10.1016/s1570-9639(02)00532-0 12535616

[69] Jin W Fuki IV Seidah NG Benjannet S Glick JM Rader DJ. Proprotein Convertases [Corrected] are Responsible for Proteolysis and Inactivation of Endothelial Lipase. J Biol Chem (2005) 280:36551–9. doi: 10.1074/jbc.M502264200 16109723

[70] Nour N Mayer G Mort JS Salvas A Mbikay M Morrison CJ. The Cysteine-Rich Domain of the Secreted Proprotein Convertases PC5A and PACE4 Functions as a Cell Surface Anchor and Interacts With Tissue Inhibitors of Metalloproteinases. Mol Biol Cell (2005) 16:5215–26. doi: 10.1091/mbc.e05-06-0504 PMC1266420 16135528

[71] Beck S Le Good JA Guzman M Ben Haim N Roy K Beermann F. Extraembryonic Proteases Regulate Nodal Signalling During Gastrulation. Nat Cell Biol (2002) 4:981–5. doi: 10.1038/ncb890 12447384

[72] Kara I Poggi M Bonardo B Govers R Landrier J-F Tian S. The Paired Basic Amino Acid-Cleaving Enzyme 4 (PACE4) is Involved in the Maturation of Insulin Receptor Isoform B: An Opportunity to Reduce the Specific Insulin Receptor-Dependent Effects of Insulin-Like Growth Factor 2 (IGF2). J Biol Chem (2015) 290:2812–21. doi: 10.1074/jbc.M114.592543 PMC4317022 25527501

[73] Duguay SJ Jin Y Stein J Duguay AN Gardner P Steiner DF. Post-Translational Processing of the Insulin-Like Growth Factor-2 Precursor. Analysis of O-Glycosylation and Endoproteolysis. J Biol Chem (1998) 273:18443–51. doi: 10.1074/jbc.273.29.18443 9660813

[74] Yuasa K Masuda T Yoshikawa C Nagahama M Matsuda Y Tsuji A. Subtilisin-Like Proprotein Convertase PACE4 is Required for Skeletal Muscle Differentiation. J Biochem (Tokyo) (2009) 146:407–15. doi: 10.1093/jb/mvp090 19520771

[75] Couture F D’Anjou F Desjardins R Boudreau F Day R. Role of Proprotein Convertases in Prostate Cancer Progression. Neoplasia N Y N (2012) 14:1032–42. doi: 10.1593/neo.121368 PMC3514743 23226097

[76] Mahloogi H Bassi DE Klein-Szanto AJP. Malignant Conversion of non-Tumorigenic Murine Skin Keratinocytes Overexpressing PACE4. Carcinogenesis (2002) 23:565–72. doi: 10.1093/carcin/23.4.565 11960907

[77] Fradet L Temmar R Couture F Belzile M Fortier P-H Day R. Evaluation of PACE4 Isoforms as Biomarkers in Thyroid Cancer. J Otolaryngol - Head Neck Surg J Oto-Rhino-Laryngol Chir Cervico-Faciale (2018) 47:63. doi: 10.1186/s40463-018-0311-x PMC6194618 30340539

[78] Mori K Kii S Tsuji A Nagahama M Imamaki A Hayashi K. A Novel Human PACE4 Isoform, PACE4E is an Active Processing Protease Containing a Hydrophobic Cluster at the Carboxy Terminus. J Biochem (Tokyo) (1997) 121:941–8. doi: 10.1093/oxfordjournals.jbchem.a021677 9192737

[79] Lusson J Vieau D Hamelin J Day R Chrétien M Seidah NG. cDNA Structure of the Mouse and Rat Subtilisin/Kexin-Like PC5: A Candidate Proprotein Convertase Expressed in Endocrine and Nonendocrine Cells. Proc Natl Acad Sci U S A (1993) 90:6691–5. doi: 10.1073/pnas.90.14.6691 PMC46998 8341687

[80] Nakagawa T Hosaka M Torii S Watanabe T Murakami K Nakayama K. Identification and Functional Expression of a New Member of the Mammalian Kex2-Like Processing Endoprotease Family: Its Striking Structural Similarity to PACE4. J Biochem (Tokyo) (1993) 113:132–5. doi: 10.1093/oxfordjournals.jbchem.a124015 8468318

[81] Essalmani R Hamelin J Marcinkiewicz J Chamberland A Mbikay M Chrétien M. Deletion of the Gene Encoding Proprotein Convertase 5/6 Causes Early Embryonic Lethality in the Mouse. Mol Cell Biol (2006) 26:354–61. doi: 10.1128/MCB.26.1.354-361.2006 PMC1317638 16354705

[82] De Bie I Marcinkiewicz M Malide D Lazure C Nakayama K Bendayan M. The Isoforms of Proprotein Convertase PC5 are Sorted to Different Subcellular Compartments. J Cell Biol (1996) 135:1261–75. doi: 10.1083/jcb.135.5.1261 PMC2121096 8947550

[83] Xiang Y Molloy SS Thomas L Thomas G. The PC6B Cytoplasmic Domain Contains Two Acidic Clusters That Direct Sorting to Distinct Trans-Golgi Network/Endosomal Compartments. Mol Biol Cell (2000) 11:1257–73. doi: 10.1091/mbc.11.4.1257 PMC14845 10749928

[84] Kalus I Schnegelsberg B Seidah NG Kleene R Schachner M. The Proprotein Convertase PC5A and a Metalloprotease are Involved in the Proteolytic Processing of the Neural Adhesion Molecule L1. J Biol Chem (2003) 278:10381–8. doi: 10.1074/jbc.M208351200 12529374

[85] Essalmani R Zaid A Marcinkiewicz J Chamberland A Pasquato A Seidah NG. *In Vivo* Functions of the Proprotein Convertase PC5/6 During Mouse Development: Gdf11 is a Likely Substrate. Proc Natl Acad Sci U S A (2008) 105:5750–5. doi: 10.1073/pnas.0709428105 PMC2299217 18378898

[86] Campan M Yoshizumi M Seidah NG Lee ME Bianchi C Haber E. Increased Proteolytic Processing of Protein Tyrosine Phosphatase Mu in Confluent Vascular Endothelial Cells: The Role of PC5, a Member of the Subtilisin Family. Biochemistry (1996) 35:3797–802. doi: 10.1021/bi952552d 8620001

[87] Lissitzky JC Luis J Munzer JS Benjannet S Parat F Chrétien M. Endoproteolytic Processing of Integrin Pro-Alpha Subunits Involves the Redundant Function of Furin and Proprotein Convertase (PC) 5A, But Not Paired Basic Amino Acid Converting Enzyme (PACE) 4, PC5B or PC7. Biochem J (2000) 346 Pt 1:133–8. PMC1220832 10657249

[88] Stawowy P Kallisch H Borges Pereira Stawowy N Stibenz D Veinot JP Gräfe M. Immunohistochemical Localization of Subtilisin/Kexin-Like Proprotein Convertases in Human Atherosclerosis. Virchows Arch Int J Pathol (2005) 446:351–9. doi: 10.1007/s00428-004-1198-7 15756593

[89] Stawowy P Blaschke F Kilimnik A Goetze S Kallisch H Chrétien M. Proprotein Convertase PC5 Regulation by PDGF-BB Involves PI3-Kinase/P70(S6)-Kinase Activation in Vascular Smooth Muscle Cells. Hypertens Dallas Tex 1979 (2002) 39:399–404. doi: 10.1161/hy0202.103000 11882580

[90] Benjannet S Rhainds D Hamelin J Nassoury N Seidah NG. The Proprotein Convertase (PC) PCSK9 is Inactivated by Furin and/or PC5/6A: Functional Consequences of Natural Mutations and Post-Translational Modifications. J Biol Chem (2006) 281:30561–72. doi: 10.1074/jbc.M606495200 16912035

[91] Essalmani R Susan-Resiga D Chamberland A Abifadel M Creemers JW Boileau C. *In Vivo* Evidence That Furin From Hepatocytes Inactivates PCSK9. J Biol Chem (2011) 286:4257–63. doi: 10.1074/jbc.M110.192104 PMC3039354 21147780

[92] Seidah NG Benjannet S Wickham L Marcinkiewicz J Jasmin SB Stifani S. The Secretory Proprotein Convertase Neural Apoptosis-Regulated Convertase 1 (NARC-1): Liver Regeneration and Neuronal Differentiation. Proc Natl Acad Sci U S A (2003) 100:928–33. doi: 10.1073/pnas.0335507100 PMC298703 12552133

[93] Benjannet S Rhainds D Essalmani R Mayne J Wickham L Jin W. NARC-1/PCSK9 and its Natural Mutants: Zymogen Cleavage and Effects on the Low Density Lipoprotein (LDL) Receptor and LDL Cholesterol. J Biol Chem (2004) 279:48865–75. doi: 10.1074/jbc.M409699200 15358785

[94] Cunningham D Danley DE Geoghegan KF Griffor MC Hawkins JL Subashi TA. Structural and Biophysical Studies of PCSK9 and its Mutants Linked to Familial Hypercholesterolemia. Nat Struct Mol Biol (2007) 14:413–9. doi: 10.1038/nsmb1235 17435765

[95] Abifadel M Varret M Rabès J-P Allard D Ouguerram K Devillers M. Mutations in PCSK9 Cause Autosomal Dominant Hypercholesterolemia. Nat Genet (2003) 34:154–6. doi: 10.1038/ng1161 12730697

[96] Zhang D-W Lagace TA Garuti R Zhao Z McDonald M Horton JD. Binding of Proprotein Convertase Subtilisin/Kexin Type 9 to Epidermal Growth Factor-Like Repeat A of Low Density Lipoprotein Receptor Decreases Receptor Recycling and Increases Degradation. J Biol Chem (2007) 282:18602–12. doi: 10.1074/jbc.M702027200 17452316

[97] Ni YG Condra JH Orsatti L Shen X Di Marco S Pandit S. A Proprotein Convertase Subtilisin-Like/Kexin Type 9 (PCSK9) C-Terminal Domain Antibody Antigen-Binding Fragment Inhibits PCSK9 Internalization and Restores Low Density Lipoprotein Uptake. J Biol Chem (2010) 285:12882–91. doi: 10.1074/jbc.M110.113035 PMC2857140 20172854

[98] Campbell RD Law SK Reid KB Sim RB. Structure, Organization, and Regulation of the Complement Genes. Annu Rev Immunol (1988) 6:161–95. doi: 10.1146/annurev.iy.06.040188.001113 2898251

[99] Law SK Dodds AW. The Internal Thioester and the Covalent Binding Properties of the Complement Proteins C3 and C4. Protein Sci Publ Protein Soc (1997) 6:263–74. doi: 10.1002/pro.5560060201 PMC2143658 9041627

[100] Misumi Y Oda K Fujiwara T Takami N Tashiro K Ikehara Y. Functional Expression of Furin Demonstrating its Intracellular Localization and Endoprotease Activity for Processing of Proalbumin and Complement Pro-C3. J Biol Chem (1991) 266:16954–9. doi: 10.1016/S0021-9258(18)55396-5 1885622

[101] Goldberger G Colten HR. Precursor Complement Protein (Pro-C4) is Converted *In Vitro* to Native C4 by Plasmin. Nature (1980) 286:514–6. doi: 10.1038/286514a0 6447255

[102] Nakayama K. Furin: A Mammalian Subtilisin/Kex2p-Like Endoprotease Involved in Processing of a Wide Variety of Precursor Proteins. Biochem J (1997) 327(Pt 3):625–35. doi: 10.1042/bj3270625 PMC1218878 9599222

[103] Chan AC Mitchell KR Munns TW Karp DR Atkinson JP. Identification and Partial Characterization of the Secreted Form of the Fourth Component of Human Complement: Evidence That it is Different From Major Plasma Form. Proc Natl Acad Sci U S A (1983) 80:268–72. doi: 10.1073/pnas.80.1.268 PMC393354 6572001

[104] Hortin G Chan AC Fok KF Strauss AW Atkinson JP. Sequence Analysis of the COOH Terminus of the Alpha-Chain of the Fourth Component of Human Complement. Identification of the Site of its Extracellular Cleavage. J Biol Chem (1986) 261:9065–9. doi: 10.1016/S0021-9258(19)84488-5 3722187

[105] Ooi YM Colten HR. Biosynthesis and Post-Synthetic Modification of a Precursor (Pro-C5) of the Fifth Component of Mouse Complement (C5). J Immunol Baltim Md 1950 (1979) 123:2494–8. 501083

[106] Nilsson SC Sim RB Lea SM Fremeaux-Bacchi V Blom AM. Complement Factor I in Health and Disease. Mol Immunol (2011) 48:1611–20. doi: 10.1016/j.molimm.2011.04.004 21529951

[107] Timár KK Junnikkala S Dallos A Jarva H Bhuiyan ZA Meri S. Human Keratinocytes Produce the Complement Inhibitor Factor I: Synthesis is Regulated by Interferon-Gamma. Mol Immunol (2007) 44:2943–9. doi: 10.1016/j.molimm.2007.01.007 17320177

[108] Barnum SR Schein TN. The Complement FactsBook. Academic Press. (2017). London, United Kingdom 514 p.

[109] Tsiftsoglou SA Sim RB. Human Complement Factor I Does Not Require Cofactors for Cleavage of Synthetic Substrates. J Immunol Baltim Md 1950 (2004) 173:367–75. doi: 10.4049/jimmunol.173.1.367 15210795

[110] Goldberger G Arnaout MA Aden D Kay R Rits M Colten HR. Biosynthesis and Postsynthetic Processing of Human C3b/C4b Inactivator (Factor I) in Three Hepatoma Cell Lines. J Biol Chem (1984) 259:6492–7. doi: 10.1016/S0021-9258(20)82168-1 6327681

[111] Ullman CG Chamberlain D Ansari A Emery VC Haris PI Sim RB. Human Complement Factor I: Its Expression by Insect Cells and its Biochemical and Structural Characterisation. Mol Immunol (1998) 35:503–12. doi: 10.1016/s0161-5890(98)00052-2 9809578

[112] Wong MJ Goldberger G Isenman DE Minta JO. Processing of Human Factor I in COS-1 Cells Co-Transfected With Factor I and Paired Basic Amino Acid Cleaving Enzyme (PACE) cDNA. Mol Immunol (1995) 32:379–87. doi: 10.1016/0161-5890(94)00151-p 7739577

[113] Zhou Y Wu Q. Corin in Natriuretic Peptide Processing and Hypertension. Curr Hypertens Rep (2014) 16:415. doi: 10.1007/s11906-013-0415-7 24407448 PMC3941447

[114] Gladysheva IP Robinson BR Houng AK Kováts T King SM. Corin is Co-Expressed With Pro-ANP and Localized on the Cardiomyocyte Surface in Both Zymogen and Catalytically Active Forms. J Mol Cell Cardiol (2008) 44:131–42. doi: 10.1016/j.yjmcc.2007.10.002 17996891

[115] Jiang J Wu S Wang W Chen S Peng J Zhang X. Ectodomain Shedding and Autocleavage of the Cardiac Membrane Protease Corin. J Biol Chem (2011) 286:10066–72. doi: 10.1074/jbc.M110.185082 PMC3060458 21288900

[116] Liao X Wang W Chen S Wu Q. Role of Glycosylation in Corin Zymogen Activation. J Biol Chem (2007) 282:27728–35. doi: 10.1074/jbc.M703687200 17660514

[117] Dong N Fang C Jiang Y Zhou T Liu M Zhou J. Corin Mutation R539C From Hypertensive Patients Impairs Zymogen Activation and Generates an Inactive Alternative Ectodomain Fragment. J Biol Chem (2013) 288:7867–74. doi: 10.1074/jbc.M112.411512 PMC3597824 23372161

[118] Gherardini PF Wass MN Helmer-Citterich M Sternberg MJE. Convergent Evolution of Enzyme Active Sites is Not a Rare Phenomenon. J Mol Biol (2007) 372:817–45. doi: 10.1016/j.jmb.2007.06.017 17681532

[119] Chen S Wang H Li H Zhang Y Wu Q. Functional Analysis of Corin Protein Domains Required for PCSK6-Mediated Activation. Int J Biochem Cell Biol (2018) 94:31–9. doi: 10.1016/j.biocel.2017.11.010 PMC5745258 29180304

[120] Dahl MR Thiel S Matsushita M Fujita T Willis AC Christensen T. MASP-3 and its Association With Distinct Complexes of the Mannan-Binding Lectin Complement Activation Pathway. Immunity (2001) 15:127–35. doi: 10.1016/s1074-7613(01)00161-3 11485744

[121] Cortesio CL Jiang W. Mannan-Binding Lectin-Associated Serine Protease 3 Cleaves Synthetic Peptides and Insulin-Like Growth Factor-Binding Protein 5. Arch Biochem Biophys (2006) 449:164–70. doi: 10.1016/j.abb.2006.02.006 16554018

[122] Gajek G Świerzko AS Cedzyński M. Association of Polymorphisms of MASP1/3, COLEC10, and COLEC11 Genes With 3MC Syndrome. Int J Mol Sci (2020) 21:E5483. doi: 10.3390/ijms21155483 32751929 PMC7432537

[123] Malfait A-M Seymour AB Gao F Tortorella MD Le Graverand-Gastineau M-PH Wood LS. A Role for PACE4 in Osteoarthritis Pain: Evidence From Human Genetic Association and Null Mutant Phenotype. Ann Rheum Dis (2012) 71:1042–8. doi: 10.1136/annrheumdis-2011-200300 PMC3603144 22440827

[124] Sirmaci A Walsh T Akay H Spiliopoulos M Sakalar YB Hasanefendioğlu-Bayrak A. MASP1 Mutations in Patients With Facial, Umbilical, Coccygeal, and Auditory Findings of Carnevale, Malpuech, OSA, and Michels Syndromes. Am J Hum Genet (2010) 87:679–86. doi: 10.1016/j.ajhg.2010.09.018 PMC2978960 21035106

[125] Dobó J Pál G Cervenak L Gál P. The Emerging Roles of Mannose-Binding Lectin-Associated Serine Proteases (MASPs) in the Lectin Pathway of Complement and Beyond. Immunol Rev (2016) 274:98–111. doi: 10.1111/imr.12460 27782318

[126] Garred P Genster N Pilely K Bayarri-Olmos R Rosbjerg A Ma YJ. A Journey Through the Lectin Pathway of Complement-MBL and Beyond. Immunol Rev (2016) 274:74–97. doi: 10.1111/imr.12468 27782323

[127] Gál P Dobó J. “MASP-1.,”. In: Choi S, editor. Encyclopedia of Signaling Molecules. Cham: Springer International Publishing (2018). p. 2965–72. doi: 10.1007/978-3-319-67199-4_101691

[128] Takahashi M Ishida Y Iwaki D Kanno K Suzuki T Endo Y. Essential Role of Mannose-Binding Lectin-Associated Serine Protease-1 in Activation of the Complement Factor D. J Exp Med (2010) 207:29–37. doi: 10.1084/jem.20090633 20038603 PMC2812541

[129] Takahashi M Iwaki D Kanno K Ishida Y Xiong J Matsushita M. Mannose-Binding Lectin (MBL)-Associated Serine Protease (MASP)-1 Contributes to Activation of the Lectin Complement Pathway. J Immunol Baltim Md 1950 (2008) 180:6132–8. doi: 10.4049/jimmunol.180.9.6132 18424734

[130] Oroszlán G Kortvely E Szakács D Kocsis A Dammeier S Zeck A. MASP-1 and MASP-2 Do Not Activate Pro-Factor D in Resting Human Blood, Whereas MASP-3 Is a Potential Activator: Kinetic Analysis Involving Specific MASP-1 and MASP-2 Inhibitors. J Immunol Baltim Md 1950 (2016) 196:857–65. doi: 10.4049/jimmunol.1501717 26673137

[131] Dobó J Szakács D Oroszlán G Kortvely E Kiss B Boros E. MASP-3 is the Exclusive Pro-Factor D Activator in Resting Blood: The Lectin and the Alternative Complement Pathways are Fundamentally Linked. Sci Rep (2016) 6:31877. doi: 10.1038/srep31877 27535802 PMC4989169

[132] Hayashi M Machida T Ishida Y Ogata Y Omori T Takasumi M. Cutting Edge: Role of MASP-3 in the Physiological Activation of Factor D of the Alternative Complement Pathway. J Immunol Baltim Md 1950 (2019) 203:1411–6. doi: 10.4049/jimmunol.1900605 31399515

[133] Lesavre PH Müller-Eberhard HJ. Mechanism of Action of Factor D of the Alternative Complement Pathway. J Exp Med (1978) 148:1498–509. doi: 10.1084/jem.148.6.1498 PMC2185104 82604

[134] Gál P Dobó J Pál G. Comment on “Cutting Edge: Role of MASP-3 in the Physiological Activation of Factor D of the Alternative Complement Pathway”. J Immunol Baltim Md 1950 (2019) 203:3091. doi: 10.4049/jimmunol.1901055 31818918

[135] Zundel S Cseh S Lacroix M Dahl MR Matsushita M Andrieu J-P. Characterization of Recombinant Mannan-Binding Lectin-Associated Serine Protease (MASP)-3 Suggests an Activation Mechanism Different From That of MASP-1 and MASP-2. J Immunol Baltim Md 1950 (2004) 172:4342–50. doi: 10.4049/jimmunol.172.7.4342 15034049

[136] Skjoedt M-O Hummelshoj T Palarasah Y Honore C Koch C Skjodt K. A Novel Mannose-Binding Lectin/Ficolin-Associated Protein is Highly Expressed in Heart and Skeletal Muscle Tissues and Inhibits Complement Activation. J Biol Chem (2010) 285:8234–43. doi: 10.1074/jbc.M109.065805 PMC2832975 20053996

[137] Iwaki D Kanno K Takahashi M Endo Y Matsushita M Fujita T. The Role of Mannose-Binding Lectin-Associated Serine Protease-3 in Activation of the Alternative Complement Pathway. J Immunol Baltim Md 1950 (2011) 187:3751–8. doi: 10.4049/jimmunol.1100280 21865552

[138] Oroszlán G Dani R Végh BM Varga D Ács AV Pál G. Proprotein Convertase Is the Highest-Level Activator of the Alternative Complement Pathway in the Blood. J Immunol Baltim Md 1950 (2021) 206:2198–205. doi: 10.4049/jimmunol.2000636 33858964

[139] Jenny L Dobó J Gál P Schroeder V. MASP-1 Induced Clotting–The First Model of Prothrombin Activation by MASP-1. PLoS One (2015) 10:e0144633. doi: 10.1371/journal.pone.0144633 26645987 PMC4672900

[140] Ramos-DeSimone N Hahn-Dantona E Sipley J Nagase H French DL Quigley JP. Activation of Matrix Metalloproteinase-9 (MMP-9) *via* a Converging Plasmin/Stromelysin-1 Cascade Enhances Tumor Cell Invasion. J Biol Chem (1999) 274:13066–76. doi: 10.1074/jbc.274.19.13066 10224058

[141] Tibbs E Cao X. Emerging Canonical and Non-Canonical Roles of Granzyme B in Health and Disease. Cancers (2022) 14:1436. doi: 10.3390/cancers14061436 35326588 PMC8946077

[142] Roumenina LT Daugan MV Petitprez F Sautès-Fridman C Fridman WH. Context-Dependent Roles of Complement in Cancer. Nat Rev Cancer (2019) 19:698–715. doi: 10.1038/s41568-019-0210-0 31666715

[143] Nitta H Murakami Y Wada Y Eto M Baba H Imamura T. Cancer Cells Release Anaphylatoxin C5a From C5 by Serine Protease to Enhance Invasiveness. Oncol Rep (2014) 32:1715–9. doi: 10.3892/or.2014.3341 25050844

[144] West EE Kolev M Kemper C. Complement and the Regulation of T Cell Responses. Annu Rev Immunol (2018) 36:309–38. doi: 10.1146/annurev-immunol-042617-053245 PMC7478175 29677470

[145] Reis ES Mastellos DC Hajishengallis G Lambris JD. New Insights Into the Immune Functions of Complement. Nat Rev Immunol (2019) 19:503–16. doi: 10.1038/s41577-019-0168-x PMC6667284 31048789

